# Aging Brain from a Network Science Perspective: Something to Be Positive About?

**DOI:** 10.1371/journal.pone.0078345

**Published:** 2013-11-06

**Authors:** Michelle W. Voss, Chelsea N. Wong, Pauline L. Baniqued, Jonathan H. Burdette, Kirk I. Erickson, Ruchika Shaurya Prakash, Edward McAuley, Paul J. Laurienti, Arthur F. Kramer

**Affiliations:** 1 Department of Psychology, University of Iowa, Iowa City, Iowa, United States of America; 2 Beckman Institute & Department of Psychology, University of Illinois at Urbana-Champaign, Urbana, Illinois, United States of America; 3 Department of Kinesiology and Community Health, University of Illinois at Urbana-Champaign, Urbana, Illinois, United States of America; 4 Department of Psychology, University of Pittsburgh, Pittsburgh, Pennsylvania, United States of America; 5 Department of Psychology, The Ohio State University, Columbus, Ohio, United States of America; 6 Wake Forest School of Medicine, Wake Forest University, Winston-Salem, North Carolina, United States of America; Emory University, United States of America

## Abstract

To better understand age differences in brain function and behavior, the current study applied network science to model functional interactions between brain regions. We observed a shift in network topology whereby for older adults subcortical and cerebellar structures overlapping with the Salience network had more connectivity to the rest of the brain, coupled with fragmentation of large-scale cortical networks such as the Default and Fronto-Parietal networks. Additionally, greater integration of the dorsal medial thalamus and red nucleus in the Salience network was associated with greater satisfaction with life for older adults, which is consistent with theoretical predictions of age-related increases in emotion regulation that are thought to help maintain well-being and life satisfaction in late adulthood. In regard to cognitive abilities, greater ventral medial prefrontal cortex coherence with its topological neighbors in the Default Network was associated with faster processing speed. Results suggest that large-scale organizing properties of the brain differ with normal aging, and this perspective may offer novel insight into understanding age-related differences in cognitive function and well-being.

## Introduction

Cognitive aging affects abilities such as processing speed and executive function abilities such as task-switching, working memory, and inhibitory control, whereas emotion regulation and life satisfaction typically increase after middle-age [1]–[3]. These higher-level cognitive abilities and emotion regulation require coordination among interacting functional brain networks. Therefore, examining the brain from a functional systems perspective is important for identifying fundamental organizational properties of the aging brain that could explain the observed divergent pattern of cognitive impairment coupled with preserved psychological well-being with aging.

Under the realm of network science, a brain graph models the brain as a complex system of interacting nodes connected by a set of edges. Nodes in brain graphs have commonly been modeled as separate anatomical regions of interest (ROIs) derived from population atlases or as voxels, the three-dimensional pixels used to represent brain images. Edges in a brain graph can either represent structural connections between nodes, such as integrity of white matter tracts, or may represent the strength of functional coupling between nodes. Edges can thus represent functional relationships regardless of physical proximity, where the strength of functional relationships are reflected by the number of edges, or topological distance, that need to be traversed to connect any given nodes. Based on these fundamental graph constructs, many rich metrics about functional communication patterns in the system can be derived. For instance, research has shown the human brain is organized as a “small-world” network, a common network interaction pattern in biological and real-world systems such as social networks, the internet and commercial air-traffic patterns [4], [5], which is characterized by a balance of highly interconnected communities of nodes that are themselves interconnected through nodes that have connections across communities [4]. The balance of integration through paths that connect otherwise unconnected communities, and segregation through smaller interconnected communities is also reflected by the typical degree distribution of small-world networks, where degree (*K*) is a measure of how many nodes any one node is connected to. Small-world networks have a degree distribution that approximates a power law and tend to be scale-free, reflecting the predominance of low-degree nodes and less probable, but critical, very high-degree nodes known as hubs.

A network’s similarity or deviance to small world characteristics can be measured by graph metrics such as clustering coefficient and characteristic path length. In the context of graph or topological space, clustering coefficient quantifies strength of local connectivity among nodes, whereas path length measures the shortest path of edges between two nodes and thus reflects their potential for fast communication. However, local and global efficiency have been proposed as more useful measures of local community clustering and fast communication throughout the network, respectively [6], [7]. For instance path length is not universally scaled and its meaning is relative to the size of the network, whereas efficiency measures are scaled from zero to 1 with zero being least efficient (fully disconnected) and 1 being most efficient (fully connected). Additionally, path length is infinite for isolated nodes, which results in mean path length being more heavily weighted towards long paths than short [8]. For these reasons, in this study we used global and local efficiency (denoted as *Eglob* and *Eloc* respectively) as measures of topological integration and segregation instead of path length and clustering coefficient.

A related network concept that characterizes the potential for information flow is the network *core*. The core of a network represents nodes that form the necessary infrastructure for the network to remain intact amidst node removal (e.g., atrophy or disconnection) [9]. To find the core, the k-core decomposition method is used, whereby nodes are sequentially removed from the network starting with degree of 1. Each node is given a core number corresponding to the largest degree *K* before it was removed without network collapse [9], [10]. Therefore, nodes with a high core number represent regions that are not only well-connected, but which are well-connected to other well-connected nodes and so form the “backbone” of the network. Thus of all nodes, high core nodes are most well-suited for spreading information (or perhaps disease) efficiently. One study using this method with diffusion imaging of white matter integrity found that the posterior cingulate was the most consistently observed core region [9].

Regarding aging, Meunier et al., [11] used atlas-based ROIs as network nodes and showed that aging was associated with fragmented long-distance communication between frontal and both temporal and posterior brain regions, coupled with decreased inter-community and intra-community hub activity in all major communities, or modules, and particularly in the frontal cortex. This complements studies using other measures to examine brain networks, which have found aging is reliably associated with less functional connectivity throughout the Default Mode Network (DMN) [12]–[16]. The DMN typically includes the posterior cingulate cortex, the dorsal, anterior, and ventral medial prefrontal cortices, the bilateral lateral occipital cortex, and the hippocampal, parahippocampal, and middle temporal cortices.

In addition to the DMN, several other association networks relevant to aging include a Fronto-parietal network, Fronto-executive network, and the Salience network. The Fronto-parietal network commonly includes the inferior parietal cortices, the supplementary motor and primary cortices, the frontal eye-fields, primary and extrastriate visual cortices, the inferior frontal cortex, and some overlapping portions of the temporo-parietal junction with the fronto-executive network; it has been associated with rapid goal-directed tuning of visual attention [17]. In contrast, the Fronto-executive network (also referred to as the cingulo-opercular network) commonly includes the anterior prefrontal cortex, frontal insular and operculum cortices, the temporo-parietal junction, the dorsal posterior cingulate, and the dorsal anterior cingulate gyrus; it has been associated with sustained maintenance of task goals and conflict monitoring [17]. Previous studies have observed age-related decrements in functional connectivity within both the Fronto-parietal and Fronto-Executive networks [13], [18], [19].

The Salience network partially overlaps with regions in the Fronto-executive network, including the dorsal anterior cingulate and frontal insula and operculum, and also includes sub-cortical, limbic, and cerebellar structures, such as the sublenticular extended amygdala, ventral stiatopallidum, dorsomedial thalamus, hypothalamus, red nucleus, periaqueductal gray, the substantia nigra/ventral tegmental area, and the Crus I region in the lateral cerebellar hemispheres [20]–[22]. Behaviorally, the Salience network has been associated with emotion processing, autonomic regulation and interoceptive awareness, and pain perception [20], [22]. The overlap of the Fronto-executive and Salience networks illustrates the presence of some brain regions such as the dorsal anterior cingulate that are involved in cognitive processes that bridge across multiple networks, and which likely play a role in the interaction of large scale networks. Thus modeling the brain as one large interacting network, as in the current study, promises to reveal insight about adult differences in brain function that are missed by only examining interactions within (regionally exclusive) brain networks in relation to behavior.

Two recent studies that examined voxel-wise functional networks observed greater degree in regions that overlap with the Salience network in middle-aged and older adults [12], [23]. These results suggest that older adults may have greater network integration in the Salience network, however neither study examined the association between network measures and individual differences in behavioral indices that would indicate whether this pattern could reflect adaptive or maladaptive aging processes. Thus, the behavioral relevance of the finding remains untested. Since the Salience network is thought to be associated with awareness of emotional significance and affective states, increases in integrity of the network are consistent with predictions of the socioemotional selectivity theory, which posits that older adults maintain affective and general well-being in late life via increased motivation to regulate emotional experience in order to optimize a sense of meaning and fulfillment [2]. The current study extends previous research on the Salience network by including multiple measures of network integration in addition to measures used previously (similar to degree), and by examining the association between network integration and life satisfaction in older adults. Age-related differences in network topology were compared with two groups matched on sex, education, and in-scanner motion, including a young adult group (N = 32, mean age = 24 years) and an older adult group (N = 30, mean age = 64 years).

Given previous research that has highlighted the trend of age-related differences in functional connectivity in large association networks that require integration across many cortical and sub-cortical sites, particularly in the DMN, we hypothesized that age differences would be largest in integration measures such as degree, global efficiency, and k-core and regionally in areas that overlap with the DMN. We expected age deficits in DMN integration metrics to relate to poorer cognitive performance. We also expected older adults to have less local efficiency, which could be widespread based on decreased processing specificity and network independence in the aging brain [18], [24], [25]. In contrast, given the observed increase in well-being and emotional regulation with age and greater integration of areas involved in emotion processing, older adults may have greater integration in regions known to be involved in emotion regulation coupled with greater emotional well-being. Hypotheses were evaluated with whole-brain (data-driven, exploratory) voxel-wise graph theoretical analyses.

## Materials and Methods

All procedures were approved by the Institutional Review Board at the University of Illinois at Urbana-Champaign.

### 2.1. Participants

Participants were recruited from the local community of Urbana-Champaign, Illinois. Eligible participants had to (1) demonstrate strong right handedness, with a 75% or above on the Edinburgh Handedness Questionnaire (Oldfield, 1971), (2) be between the ages of 18 and 35 for young adults and between 55 and 80 years for elderly adults (3) score >51 on the modified Mini-Mental Status Exam (mMMSE, Stern et al., 1987), a screening questionnaire to rule out potential neurological pathology, (4) score <3 on the Geriatric Depression Scale (GDS) [26], (5) have normal color vision (6) have a corrected visual acuity of at least 20/40 and (7) sign an informed consent. Participants completed a mock MRI session, wherein they were screened for their ability to complete an experiment in an MRI environment. Participants who passed the mock screening subsequently completed a series of structural and functional MRI scans. Prior to MR scanning, all participants were tested for visual acuity and (if need be) corrective lenses were provided within the viewing goggles to ensure a corrected vision of at least 20/40 while in the scanner. Participants were compensated for their participation.

Demographic data are presented in Table 1. Data from the full sample have been reported in previous studies from our lab [16]. In this study, for the network topology group comparison, an Old adult sub-sample was formed with approximately the same number of participants, gender composition, and absolute and relative average head displacement as the young adults. Priority was given to average relative head displacement (mm) in matching participants on movement since frame-wise displacement relative to the image from the previous time-point (TR) is closely aligned to movement-related changes in BOLD signal [27], [28]; the overlap of relative displacement frequency distributions for young and older adult groups before and after motion-matching is visualized in figure S6. Neuroimaging measures were collected as part of a larger task battery, and were originally developed to be passive viewing tasks for localizing stimulus-specific processing regions of the ventral visual cortex [29], [30].

**Table 1:** Demographics, cognitive status, and motion characteristics.

Variable	Young AdultControl	Older Adultsample	Older Motionmatched group	YA -vs-OA	YA-vs-OA matched
N	32	120	30		
Age (SD)^ a^	23.9 (4.4)	66.5 (5.7)	64.1 (2.7)	***	***
% Female	85	71	83	NS	NS
Education^b^	16.8 (2.1)	15.8 (3.0)	15.9 (3.1)	NS	NS
mMMSE^c^	*na*	54.8 (1.9)	55.1 (1.6)	*na*	*na*
Average absolutemotion (mm)^c^ (SD)	.273 (.22)	.436 (.25)	.295 (.10)	***	NS
Relative motion(mm) (SD)	.0975 (.03)	.1516 (.06)	.0902 (.01)	***	NS

**Notes.** ^a^Age ranges were Young Adult Control (19–35 yrs), Older Adult sample (59–79 yrs), and Older Motion matched group (59–78 yrs);

b Education refers to self-reported years of education;

c a modified MMSE was given, with a maximum score of 52;

c absolute head displacement was measured with reference to the middle volume of each functional run;

* p<.05,

** p<.01,

*** p<.001, NS p>.05;

*na* indicates relevant data was not collected.

### 2.2. Measures

All participants in the study completed a battery of neuropsychological tests. Cognitive measures were included based on sensitivity to individual differences in aging and their availability for both young and older adults.

#### 2.2.1. Spatial working memory

This task required formation and maintenance of the locations of several items on a display, providing a measure of working memory. First, a fixation crosshair appeared for one second and participants were instructed to keep their eyes on the crosshair. Following the fixation, either one, two, or three black dots appeared at random locations on the screen for a duration of 500 ms. The dots were removed from the display and the fixation cross re-appeared on the screen for a period of three seconds. During this time, participants were instructed to try and remember the locations of the previously presented black dots. At the end of the three-second delay, a red dot appeared on the screen in either one of the same locations as the target dots (match condition) or at a different location (nonmatch condition). Participants had two seconds to respond to the red dot by pressing one of two keys on a standard keyboard – the ‘x’ key for a nonmatch trial, and the ‘m’ key for a match trial. Forty trials were presented for each set size (1, 2, or 3 locations), with 20 trials as match trials and 20 trials as nonmatch trials. Participants were instructed to respond as quickly and accurately as possible. Several practice trials were performed before the task began in order to acquaint the participants with task instructions and responses. Since our goal was to characterize general spatial working memory performance, we computed a composite variable of average mean reaction time and accuracy across all three conditions.

#### 2.2.2. Task-Switching

This task provided a measure of processing speed and executive function by testing participants’ abilities to a) respond quickly and accurately under a single task set, and b) flexibly switch focus of attention between multiple task sets. In the task-switching block participants had to switch between judging whether a number (1, 2, 3, 4, 6, 7, 8, or 9) was odd or even and judging whether it was low or high (i.e., smaller or larger than 5), whereas in the single task block participants completed one task sequentially over all trials. Numbers were presented individually for 1500 ms against a pink or blue background at the center of the screen, with the constraint that the same number did not appear twice in succession. If the background was blue, participants used one hand to report as quickly as possible whether the letter was high (“X” key) or low (“Z” key). If the background was pink, participants used their other hand to report as quickly as possible whether the number was odd (“N” key) or even (“M” key). Participants completed four single task blocks (2 blocks of odd/even and 2 blocks of high/low) of 24 trials each. Due to the difficulty of this task, participants were provided with a practice block in which they switched from one task to the other for 120 trials. Finally, they completed a dual task (switching) block of 120 trials during which the task for each trial was chosen randomly. For the current study, the primary dependent measures of interest were a measure of processing speed from single task speed and accuracy, and executive function measures of switch cost. Switch cost was measured as either local or global cost. Local cost was computed as the mean reaction time difference between switch and non-switch trials, and is a measure of attentional set re-configuration and inhibition. Global cost was computed as the mean reaction time difference between mixed and single task blocks, and is a measure of the attentional overhead resulting from maintenance of two task sets.

#### 2.2.3. General well-being

Subjective well-being was assessed with the five-item Satisfaction With Life Scale (SWLS) [31]. Life satisfaction is thought to be a cognitive-judgmental dimension of well-being that is more stable than positive and negative affect judgments [31], [32]. Studies have demonstrated that the scale has good psychometric properties with young and older adults [32]. Participants rated the following statements on a 7-point likert scale of 1-Strongly disagree to 7-Strongly agree: in most ways my life is close to ideal; the conditions of my life are excellent; I am satisfied with my life; so far I have gotten the important things I want in life; if I could life my life over, I would change almost nothing. Ratings were summed to generate a range of scores from 5 to 35. Only the older adults in this study completed the survey; data was not available for 1 participant.

### 2.3. Imaging Methods

#### 2.3.1. Structural MRI

For all participants, high resolution (1.3 mm×1.3 mm×1.33 mm) T1-weighted brain images were acquired using a 3D MPRAGE (Magnetization Prepared Rapid Gradient Echo Imaging) protocol with 144 contiguous axial slices, collected in ascending fashion parallel to the anterior and posterior commissures, echo time (TE) = 3.87 ms, repetition time (TR) = 1800 ms, field of view (FOV) = 256 mm, acquisition matrix 192 mm×192 mm, and flip angle = 8°. All images were collected on a 3T head-only Siemens Allegra MRI scanner.

#### 2.3.2. Functional MRI

Functional MRI (fMRI) scans were acquired during three passive viewing tasks: 1) a checkerboard task comprised of luminance-matched flashing black-and-white checkerboards and flashing color checkerboards at a rate of 8 Hz, each checkerboard condition was presented in two separate 30-second blocks that alternated with 20-second blocks of fixation baseline; 2) a word viewing task comprised of 30-second blocks of words, pseudo-words, and letter strings, presented separately in two 30-second blocks that alternated with 20-second blocks of fixation baseline, each block consisted of 20 unique stimuli that were each presented for one-second with a 500-ms fixation between each word presentation; and 3) a face/building viewing task comprised of three 20-second blocks of faces and buildings that alternated with 20-second blocks of luminance matched scrambled images (taken from the face and building stimulus set) as the baseline condition, each block consisted of 20 unique black-and-white images (controlled for luminance and dimension) that were each presented for one-second. In each task participants were instructed to keep their eyes open and to pay attention to the screen.

Visual stimuli were presented with MRI-safe fiber optic goggles (Resonance Technologies, Inc.). Participants completed the passive viewing tasks as part of a larger battery of cognitive paradigms within the scanner. For the fMRI tasks, low resolution (3.44 mm×3.44 mm×4 mm) T2* weighted images were acquired using a fast echo-planar imaging (EPI) sequence with Blood Oxygenation Level Dependent (BOLD) contrast (64×64 matrix, TR = 1500 ms, TE = 26 ms, flip angle = 60). A total of 150 volumes were acquired per participant for the checkerboard task, 220 volumes for the word task, and 180 volumes for the face/building task.

### 2.4. Image Analysis

#### 2.4.1. Structural MRI preprocessing

Each participant’s low-resolution EPI image was registered to his or her high-resolution T1 structural image, which was subsequently registered to stereotaxic space (study-specific template generated using 152 T1 MNI as the target volume, Montreal Neurological Institute) using FLIRT 12-parameter affine linear registration [33]. A study-specific template was made from the structural images of the adults in this sample. To make the study-specific template, high-resolution structural images were first skull-stripped using BET [34], and manually inspected and corrected for any skull-stripping errors. Next, the structural images were registered to the 152 T1 MNI volume using FLIRT 12-parameter affine linear registration [33]. Finally, registered volumes were averaged to form a representative reference volume. Before group analyses, functional data were registered to stereotaxic space using transforms generated from the alignment of high-resolution T1 images.

#### 2.4.2. Preprocessing of functional data

Detailed description of the functional connectivity procedures are reported elsewhere [16], [18], which followed standard procedures for functional connectivity preprocessing [13], [35]. In addition to the typical nuisance regression of white matter, CSF, and global signal, to further isolate our examination to intrinsic functional connectivity, we also controlled for signal from a bilateral ROI in primary visual cortex (125 anatomical-voxel spheres centered at ±18, −98, −4, derived from the literature [13]). This visual cortex regressor, along with the global signal regressor, were procedures to ensure our estimates of functional connectivity were not inflated due to the additive influence of synchronized task-evoked signal change. In addition to nuisance fMRI signal, six motion parameters computed by rigid body translation and rotation in preprocessing [33] were included in the nuisance regression. Next, validated procedures outlined in the literature were followed for concatenating the three functional runs into a single functional volume [36], which are summarized in figure S4. To increase the efficiency of the computationally intensive network analyses, after registration to 2 mm isotropic MNI space functional images were down-sampled to 4 mm isotropic voxel space. The brain networks were constructed with each voxel serving as node [12], [23], [37].

#### 2.4.3. Creating brain networks

The specific methods for this analysis are detailed elsewhere [37], and here we highlight the general steps and measures for applying these methods to the current project.

Following preprocessing, Pearson correlation coefficients were calculated between each voxel node and all possible combinations of voxel node pairs. We constricted analysis to primarily gray matter voxels, as determined by a mask of the average gray matter space across all subjects (∼30,000 voxels per functional brain in this data). The average gray matter mask was computed as the top 80% of intensity values in FSL’s standard space gray matter tissue prior (see figure S5 for a figure of mask coverage). Next, each correlation matrix was thresholded to keep only the strongest associations (see below), and converted to a binary voxel x voxel adjacency matrix with 1 indicating the presence and 0 indicating the absence of a connection between two voxel nodes. Thresholds were set to ensure comparable networks across subjects, as done in previous studies with this method [37].

That is, in order to compare data across participants, it was necessary to generate networks with comparable density. To achieve this, the networks were defined so that the relationship between the number of nodes *N* and the average node degree *K* (i.e., number of connections per node) was the same across all subjects. Specifically, the network was defined so that *S* = log(*N*)/log(*K*) was the same across participants. This relationship is based on the expected path length of an Erdös-Rènyi random network with *N* nodes and average degree *K* [4], [38], [39]. In an undirected, binary network as in the current study, path length refers to the number of links or edges between two nodes in topological space, and since the path length of a network with *N* and *K* is shortest when the network is Erdös-Rènyi, *S* can also be described as the theoretical lower bound of the path length. This procedure does not determine the path length of a network, but controls for connection density and ensures that for a given network size the average degree will be the same across subjects. In the current study we present results from thresholds of *S* = 2.5 and *S* = 3.0, which have been used in previous studies with this method [37], [40], [41] demonstrated the stability in matching across subjects (see supplementary materials, Figure S9).

#### 2.4.4. Network measures of brain systems

From the thresholded adjacency matrix, derived network measures included: global and local efficiency, degree, and network core. We describe each of these below.

Global and local efficiency quantify the efficiency of integrative and tight neighboring communication patterns, respectively [8]. Global and local efficiency metrics are normalized on a scale from zero to 1, with zero being least efficient and 1 being most efficient. It is thought that high global efficiency reflects greater capacity for fast, long-distant communication since a shorter path between two nodes allows less chance for noise to interfere or alter communication between nodes [8]. Specifically, node global efficiency E_glob_(*i*) was calculated as:
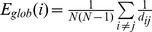
where *d*_ij_ is the shortest topological distance or the smallest number of edges (i.e., shortest path) between nodes *i* and *j*. Distance between all possible node pairs in the thresholded network was calculated by Dijkstra’s algorithm [42] as implemented in the MatlabBGL package (David Gleich; Stanford University, Stanford, CA). In sum, a voxel will have high global efficiency if it has a short path to many other voxels.

Nodal local efficiency (*Eloc*) is calculated as the global efficiency between nodes in a sub-graph including all direct topological neighbors of the node but excluding the node itself. A voxel will have high local efficiency if each of its topological neighbors (i.e., where path length = 1) also have short paths between themselves (e.g., your friends tend to be friends with each other, independent of physical proximity).

Degree is a measure of the number of edges connecting a node to the rest of the network [8]. Thus a node with high degree has many “friends” in the brain that are just one step away, and this is thought to facilitate long-distance integration of local, specialized processing modules.

To compute a measure of the extent to which a node is a network core, nodes were sequentially removed from the network starting with *K* = 1 and progressed until the network collapsed. Each node was given a core number reflecting the largest value *K* such that the node was still in the network.

Each of these measures (*Eglob*, *Eloc*, *K*, *core*) were computed for each voxel node separately [43] and projected back into brain space to visualize spatial maps of network architecture on the brain. To find the most consistent patterns of network topology across participants overlap maps were computed that reflect how often a voxel had a high network measure relative to other voxels in the map across participants. This was done by identifying voxels in the top 20% of all voxels in the map for a given network metric, for each participant, and then computing the percentage of subjects for which a given voxel was in the top 20%. For example, a voxel with an overlap value of 60 on a brain map visualizing group patterns in *K* would mean that that voxel was in the top 20% of individual-level map degree values for 60% of the participants. In this study, all mean network maps are shown with a threshold of 50% overlap. A cut-off of 20% was chosen for its consistency in replicating known hubs in group maps of young adults, and since a similar cut-off (15%) has been used in other studies with this method [40], [41].

#### 2.4.5. Measuring age-related differences in networks

To measure age-related differences in network topology difference maps were computed between overlap maps of each age group. Differences were considered “consistent” enough to interpret if there was a greater than 25% difference in the percentage of subjects that showed overlap in a given voxel. Post-hoc region-of-interest (ROI) analyses were conducted to statistically test group differences in regional graph metrics.

#### 2.4.6. Linking network measures to individual differences in performance

The relationship between individual differences in network topology and cognitive performance was assessed with a step-wise multiple linear regression, with age group and sex entered first, followed by mean-centered regional network metrics and the interaction between age group and regional network metrics, entered as independent variables; cognitive performance variables were entered as the dependent variable in separate regressions for each task, including single task (choice) response time, switch-task response time, spatial working memory response time, and spatial working memory accuracy. These analyses focused on global and local efficiency since they are similarly scaled and normally distributed between-subjects, and provide summary metrics for network integration and segregation. For models predicting task-switching speed and spatial working memory speed of response, we controlled for processing speed by also entering single task response time in the first step as a covariate. To help control for false positives and to remain conservative with regard to arbitrary network thresholding, we considered an association statistically meaningful only if the regression parameters from threshold of both S = 2.5 and S = 3.0 were statistically significant at p<.05.

#### 2.4.7. Linking network measures to individual differences in well-being in older adults

We examined the prediction that network integration in areas involved with emotion regulation would be associated with greater general well-being by correlating regional global efficiency with individual differences in SWLS scores. SWLS was only administered to older adults, therefore this analysis was only carried out with the older adult group.

## Results

Below we first explore age differences in the nodal topology of the network, followed by examination of relationships between regional variation in network metrics and performance.

### 3.1. Age Differences in Nodal Network Topology

**Figure 1:**
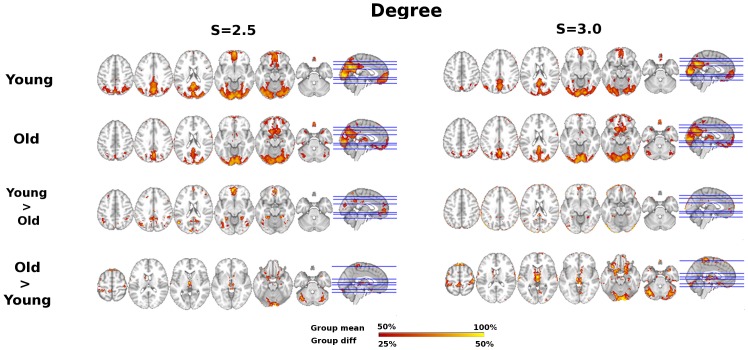
Age differences in Degree (K). Degree age group overlap maps and group differences at S = 2.5 and S = 3.0 thresholds. Sagittal slice shown at MNI X = 0; axial slices for Young, Old, and Young>Old are Z = 46, 36, 20, −10, −16, −30 shown left to right; axial slices for Old>Young are Z = 60, 16, 4, −4, −20, −30 shown left to right.

**Figure 2:**
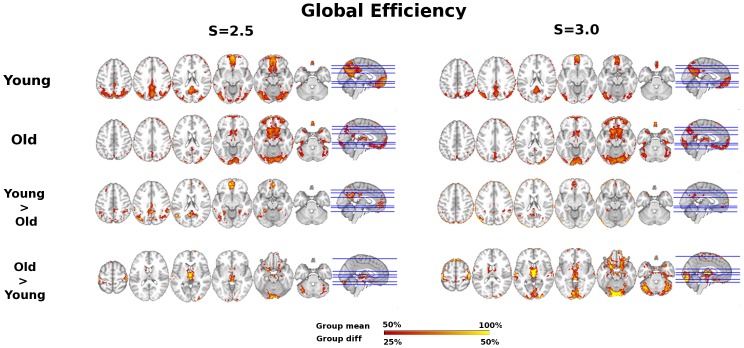
Age differences in Global Efficiency (Eglob). Global efficiency age group overlap maps and group differences at S = 2.5 and S = 3.0 thresholds. Sagittal slice shown at MNI X = 0; axial slices for Young, Old, and Young>Old are Z = 46, 36, 20, −10, −16, −30 shown left to right; axial slices for Old>Young are Z = 60, 16, 4, −4, −20, −30shown left to right.

**Figure 3:**
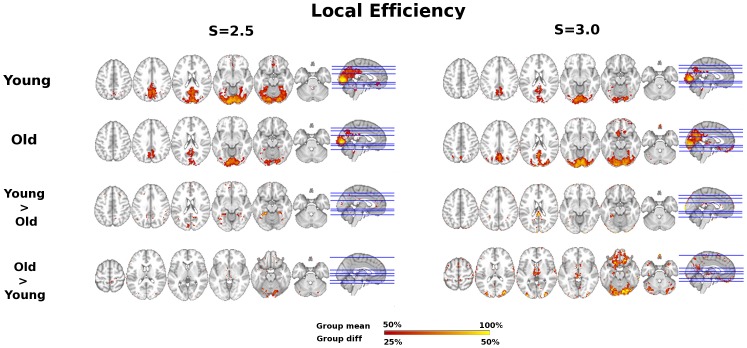
Age differences in Local Efficiency (Eloc). Local efficiency age group overlap maps and group differences at S = 2.5 and S = 3.0 thresholds. Sagittal slice shown at MNI X = 0; axial slices for Young, Old, and Young>Old are Z = 46, 36, 20, −10, −16, −30 shown left to right; axial slices for Old>Young are Z = 60, 16, 4, −4, −20, −30 shown left to right.

**Figure 4:**
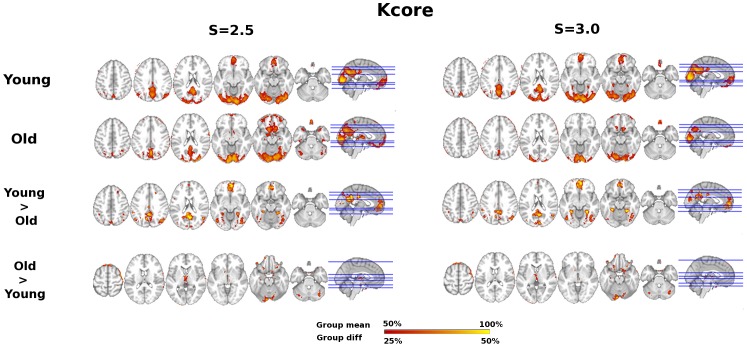
Age differences in Kcore. Kcore age group overlap maps and group differences at S = 2.5 and S = 3.0 thresholds. Sagittal slice shown at MNI X = 0; axial slices for Young, Old, and Young>Old are Z = 46, 36, 20, −10, −16, −30 shown left to right; axial slices for Old>Young are Z = 60, 16, 4, −4, −20, −30 shown left to right.

Group summary and age difference maps for network topology are visualized for *K, Eglob*, *Eloc,* and *Kcore* at thresholds S = 2.5 and S = 3.0 in Figures 1, 2, 3, and 4 respectively. The maps indicate consistency in the regions that show reduced network integration in older adults. In accord with our predictions, compared to young adults older adults have less integration in large-scale cortical networks including the DMN and the Fronto-Parietal network. On the other hand, older adults had stronger integration in the dorsal medial thalamus, red nucleus, and Crus I in the cerebellum, regions that overlap with the Salience network. Notably, the medial thalamus cluster also appears to include the midline thalamic nuclei.

**Figure 5:**
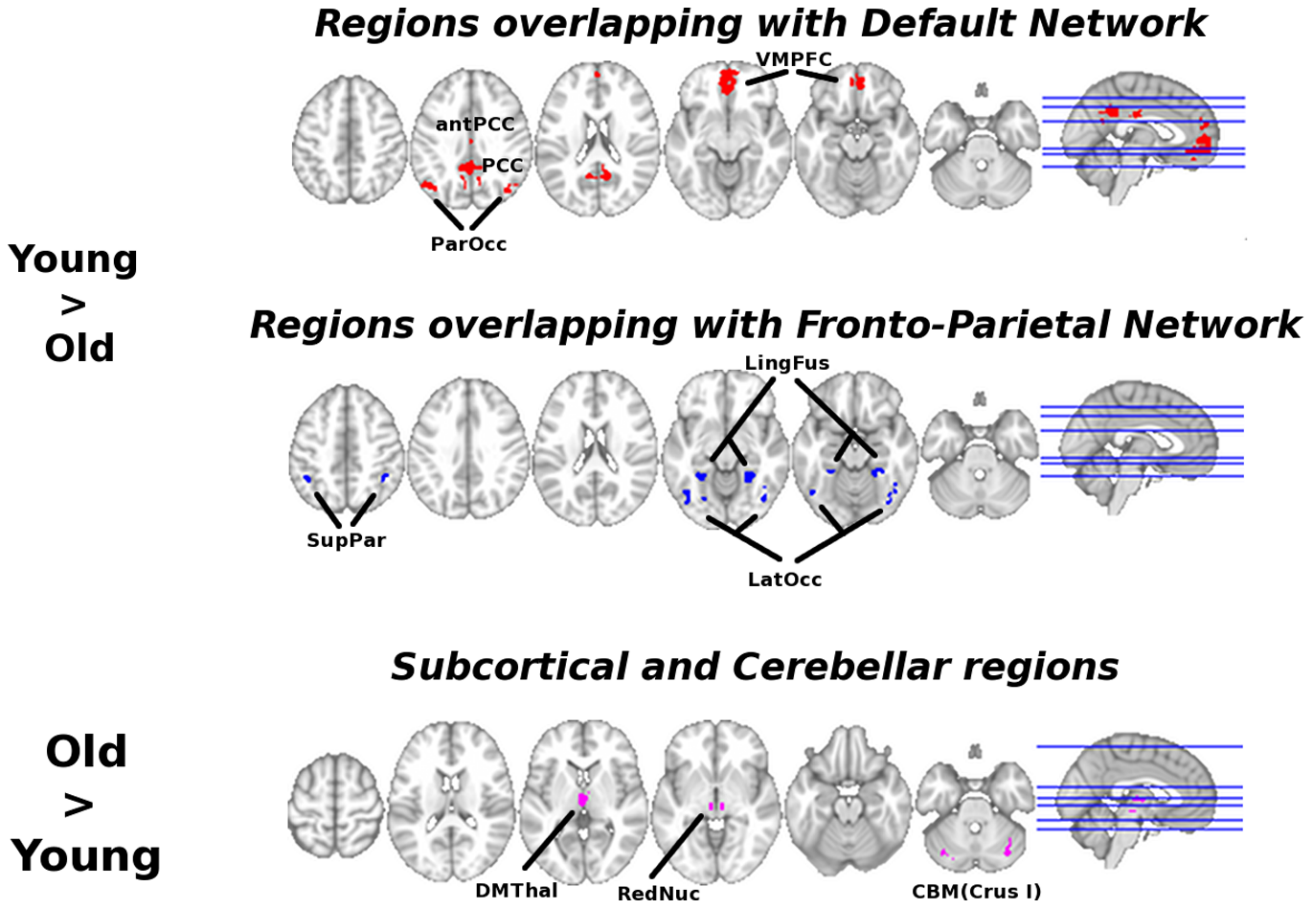
Consistent age differences in integration. Axial slices for Young>Old ROIs are Z = 46, 36, 20, −10, −16, −30 shown left to right; axial slices for Old>Young ROIs are Z = 60, 16, 4, −4, −20, −30 shown left to right. These are regions that overlapped for S = 2.50 threshold. Images shown in neurological convention, L = L, R = R.

To characterize the consistency in network integration maps (*K, Eglob, Kcore*), regions of interest were created from the conjunction of age contrast maps at threshold S = 2.5. Ten regions of interest (ROIs) resulted that are illustrated in Figure 5. Note from comparison with Figure 3, that regions with overlap map differences in local efficiency form a subset of regions with consistent network integration age differences, suggesting age differences are most prominent in regions that typically have both high local and global efficiency. Age differences in integration and local efficiency were analyzed by focusing on mean global and local efficiency (respectively) in the ten ROIs. To test for age group differences, we conducted Multivariate Analyses of Variance (MANOVA); to test for differences in the pattern of age differences across regions, we conducted repeated measures analysis across all ROIs with age group as the between-subjects factor.

Regions of interest from the default network (DMN), fronto-parietal network (FP) and from the Old>Young contrast were entered into separate MANOVAs. At S = 2.5, global efficiency results for the DMN ROIs showed a significant effect of age group, *V* = .33, F(4,57) = 7.06, p<.001, η_p_^2^ = .33; for the FP network, there was a significant effect of age group, *V* = .25, F(3,58) = 6.29, p = .001, η_p_^2^ = .25; for regions in the Old>Yng contrast, there was a significant effect of age group, *V* = .24, F(3,58) = 5.95, p = .001, η_p_^2^ = .24. At S = 2.5, local efficiency results for the DMN showed a significant effect of age group, *V* = .49, F(4,57) = 14.13, p<.001, η_p_^2^ = .49; for the FP network, there was a significant effect of age group, *V* = .45, F(3,58) = 15.71, p = .001, η_p_^2^ = .45; for regions in the Old>Yng contrast, there was a significant effect of age group, *V* = .27, F(3,58) = 7.07, p<.001, η_p_^2^ = .27. P-value and η^2^ for post-hoc univariate ANOVAs for each ROI are shown in Table 2.

**Table 2:** Summary of age effects in nodal topology of the network for S = 2.5.

S = 2.5	*Cognitive Network*	*Eglob*		*Eloc*	
*Contrast*		*p-value*	*η^2^*	*p-value*	*η^2^*
***Yng>Old***					
Posterior Cingulate	DMN	<.001	.21	<.001	.25
A. Posterior Cingulate	DMN	.002	.15	.001	.18
Ventral PFC	DMN	<.001	.27	<.001	.47
Parietal Occipital	DMN	<.001	.29	<.001	.29
Superior Parietal	FP	<.001	.20	<.001	.33
Lateral Occipital	FP	<.001	.21	<.001	.21
Lingual Fusiform	FP	<.001	.22	<.001	.34
***Old>Yng***					
DM Thalamus	SAL	.001	.17	.097	.05
Red Nucleus	SAL	<.001	.19	.005	.12
Cerebellum	SAL	.16	.03	.027	.08

**Notes.** Regions of interest from the default mode network (DMN), fronto-parietal network (FP) and Salience network (SAL) from the Old>Young contrast were entered into separate Multivariate Analyses of Variance (MANOVA); all MANOVAs were statistically significant overall (all p<.01). P-value and eta squared for post-hoc univariate ANOVAs for each ROI are shown. Group differences illustrated in Figure 6.

Results were unchanged for threshold of S = 3.0. Global efficiency results for the DMN ROIs showed a significant effect of age group, *V* = .32, F(4,57) = 6.78, p<.001, η_p_^2^ = .32; for the FP network, there was a significant effect of age group, *V* = .27, F(3,58) = 7.14, p<.001, η_p_^2^ = .27; for regions in the Old>Yng contrast, there was a significant effect of age group, *V = *.18, F(3,58) = 4.28, p = .008, η_p_^2^ = .18. For local efficiency, results for the DMN ROIs showed a significant effect of age group, *V = *.51, F(4,57) = 14.91, p<.001, η_p_^2^ = .51; for the FP network, there was a significant effect of age group, *V* = .40, F(3,58) = 13.08, p<.001, η_p_^2^ = .40; for regions in the Old>Yng contrast, there was a significant effect of age group, *V* = .29, F(3,58) = 7.79, p<.001, η_p_^2^ = .29. P-value and η^2^ for post-hoc univariate ANOVAs for each ROI are shown in Table 3.

**Table 3:** Summary of age effects in nodal topology of the network for S = 3.0.

S = 3.0	*Cognitive Network*	*Eglob*		*Eloc*	
*Contrast*		*p-value*	*η^2^*	*p-value*	*η^2^*
***Yng>Old***					
Posterior Cingulate	DMN	<.001	.23	<.001	.25
A. Posterior Cingulate	DMN	.001	.18	.003	.14
Ventral PFC	DMN	<.001	.28	<.001	.47
Parieto-Occipital	DMN	<.001	.30	<.001	.25
Superior Parietal	FP	<.001	.23	<.001	.21
Lateral Occipital	FP	<.001	.22	<.001	.20
Lingual Fusiform	FP	<.001	.23	<.001	.26
***Old>Yng***					
DM Thalamus	SAL	.005	.12	.358	.01
Red Nucleus	SAL	.002	.15	.002	.15
Cerebellum	SAL	.202	.03	.003	.13

**Notes**: Regions of interest from the default mode network (DMN), fronto-parietal network (FP) and Salience network (SAL) from the Old>Young contrast were entered into separate Multivariate Analyses of Variance (MANOVA); all MANOVAs were statistically significant overall (all p<.01). P-value and eta squared for post-hoc univariate ANOVAs for each ROI are shown. Group differences illustrated in Figure 6.

**Figure 6:**
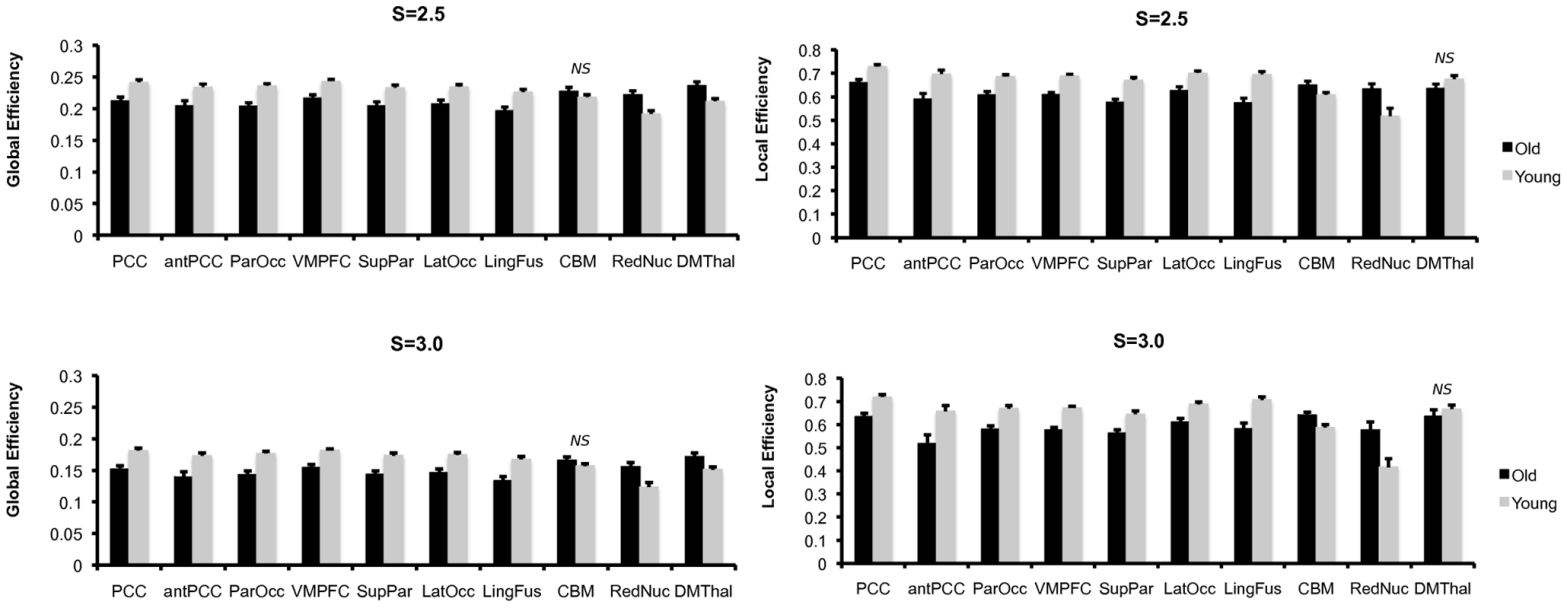
Age group differences in global and local efficiency. Group comparisons for all regions of interest were statistically significant based on post-hoc univariate ANOVAs at p<.05, except for those marked *NS*, see Tables 2 and 3. Note ROIs were generated based on an overlap of network integration measures (degree, global efficiency, and k-core); we illustrate group differences in local efficiency for comparison.

As shown in Figure 6, group differences in network metrics across ROIs differed depending on ROI location. ROIs in the DMN and FP networks showed greater global and local efficiency in favor of young adults; this provides converging statistical results with the overlap maps. For global efficiency, the red nucleus and dorsal medial thalamus were greater for older adults compared to young (see Figure 6). Repeated measures analysis indicated a significant ROI*group interaction for S = 2.5, F(9, 540) = 28.78, p<.001, η_p_^2^ = .32, and S = 3.0, F(9, 540) = 26.81, p<.001, η_p_^2^ = .31. For local efficiency, the cerebellar and red nucleus ROIs were greater for older adults compared to young (see Figure 6). Repeated measures analysis indicated a significant ROI*group interaction for S = 2.5, F(9, 540) = 11.14, p<.001, η_p_^2^ = .16, and S = 3.0, F(9, 540) = 10.73, p<.001, η_p_^2^ = .15.

### 3.2. Relationship of Network Measures to Performance

We considered an association statistically meaningful only if the regression parameters from threshold of both S = 2.5 and S = 3.0 were statistically significant at p<.05. One brain-behavior association survived this criterion, such that greater local efficiency in the ventral medial prefrontal cortex (VMPFC) was associated with faster single task (choice) response time, β = −.39, p<.05, for both S = 2.5 and S = 3.0. Older adults were generally slower and this increased the range in RT, however slopes were not statistically different between age groups (p>.05). Note when age groups were analyzed with separate regressions, with age and sex entered first, and VMPFC local efficiency entered second, the relationship between VMPFC local efficiency and single task RT was only significant for older adults, for both S = 2.5 (β = −.40, p<.05) and S = 3.0 (β = −.43, p<.05). This suggests older adults contributed more strongly than young adults to the overall negative relationship between VMPFC local efficiency and single task RT. See supplementary materials for a full listing of standardized beta parameters for each regressor and step-wise R^2^ statistics (Tables S1, S2, S3, S4, S5, S6, S7, S8, S9, S10, S11, S12).

### 3.3. Relationship of Network Measures to Well-being

**Figure 7:**
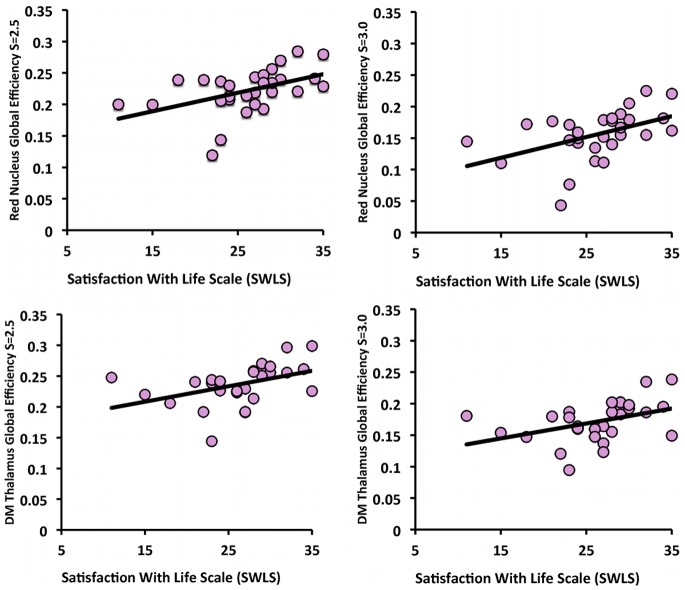
Global efficiency and life satisfaction in older adults. Scatter plots illustrating the association between global efficiency and life satisfaction for the red nucleus and dorsal medial thalamus.

The mean SWLS score for the older adults in this sample was 26.2 (SD = 5.5, range 11–35). There were no gender differences (p = .22, two-tailed), and SWLS was not significantly correlated with age or years of education (p = .38 and p = .10, respectively). Next, we correlated SWLS with *Eglob* for regions that had greater network integration based on this metric for older adults compared to young, the red nucleus and dorsal medial thalamus (see Figure 7). All correlations are reported with two-tailed p-values. Regardless of network threshold, greater SWLS was positively correlated with *Eglob* in the red nucleus (S = 2.5, r = .47, p = .01; S = 3.0, r = .47, p = .01) and dorsal medial thalamus (S = 2.5, r = .43, p = .02; S = 3.0, r = .41, p = .03), see Figure 7. SWLS was not correlated with *Eglob* at either threshold in any of the regions where older adults had less network integration compared to young (all p>.05). SWLS was not correlated with cognitive performance (all p>.05).

## Discussion

### 4.1. Age Differences in Network Topology

Given previous literature about the functional disconnection of the DMN in aging [13], [14], including replication of these studies in the current data set [16], [18], and the overlap of network hubs with the DMN [44], it is not surprising that results in favor of greater functional connectivity among several dimensions (*Degree, Eglob*, *K-core*) converge on age-related functional disruption of the DMN (see Figures 1, 2, 4, 5). However, to our knowledge this is the first study to demonstrate spatial clustering of converging network metrics for age-related differences in the DMN with voxel scale network analysis. Importantly, age differences in favor of Young also appeared to overlap with a Fronto-Parietal network, a well-replicated network in neuroimaging studies that has been associated with transient tuning of goal-directed attention. Other studies have reported age-related reductions in the functional connectivity of this network using univariate connectivity analyses [13], [16], and one study showed fragmentation of modules that overlapped with these networks in a network analysis with atlas-based ROI nodes [11]; however here we show this pattern is also evident in a data-driven network modeled at the voxel scale.

In addition, we present converging network metrics that support sub-cortical, limbic, and cerebellar structures overlapping with the Salience network, including the dorsomedial thalamus, red nucleus, and the cerebellar Crus I region, differ in network architecture as a function of age. These results are consistent with recent studies that also used a voxel-wise correlation-based network metric for characterizing voxel-wise degree [12], [23]. However, the large majority of subjects in these studies were under 60 years of age and the studies did not include behavioral measures. The dorsomedial thalamus gates information transfer between different cortical regions; its primary inputs are from the prefrontal cortex, olfactory cortex, and limbic structures such as the amygdala, and its primary output is to the prefrontal cortex via the genu of the internal capsule [45]. As noted in the Results, the midline thalamic nuclei appear to be included in the cluster we label as dorsomedial thalamus. Previous studies examining the Salience network have not included midline thalamic nuclei, however, animal studies have implicated this region as a pathway of communication between limbic and cognitive systems [46], [47]. It will be important for future research to differentiate the contribution of each of these thalamic regions to the Salience network. Both the red nucleus and Crus I cerebellar region have been implicated in salience detection and emotion processing [48], [49], supporting their involvement in the Salience network [20]. In addition, we computed supplementary post-hoc functional connectivity seed maps between each of the ROIs for the Old>Young contrast and the rest of the brain. Figures S2 and S3 show there was indeed overlap between the functional maps derived from each of these three seed regions and the Salience network described by Seeley and colleagues ([20], see also [21]).

Our results therefore suggest that late life is associated with greater brain network integration of regions in the Salience network, which may be related to the network’s role in perceiving, integrating, and responding to emotional stimuli. This “use-dependent” interpretation would be consistent with the hypothesis that older adults maintain everyday use of emotion regulation processes in order to optimize general well-being and life satisfaction in the seventh and eighth decades of life [3], [50], [51], and this is one possible explanation for the positive correlations between global efficiency in the dorsal medial thalamus and red nucleus with life satisfaction in older adults (see Figure 7). The empirical basis of this interpretation could be strengthened in future studies, because we did not directly assess self-reported emotion regulation, participants with a GDS of 3 or more were excluded (potentially limiting the range of satisfaction with life), and we did not collect a measure of well-being in young adults for comparison. Examination of these predictions in the context of additional psychological constructs hypothesized to be associated with the Salience network across the lifespan will also be important. Further, it is interesting that there were no cortical regions in the Salience network in Old>Young comparisons. We do not believe this is an artifact of potential contamination of motion, as these results are consistent with another study that corrected for relative motion between functional volumes at the individual level [12]. Thus the reason for this is unclear, but again highlights the need for more research examining age-related differences in the Salience network.

An alternate interpretation of the regions in the Salience network showing greater network integration for old compared to young adults would be degradation of the functional specificity of these brain regions. For instance, one proposed mechanism for cognitive aging is age-related decline in dopamine signaling, which is hypothesized to dampen the signal-to-noise ratio of cortico-thalamic pathways [52]. Greater network integration of the dorsomedial thalamus could represent decline in specificity of cortico-thalamic-cortical loops, resulting in noisy integrative processing in the prefrontal cortices. There is also evidence for increased iron deposition in the red nucleus with aging [53], which may impair its specificity as a relay center in motor coordination, emotional salience, and executive function networks [49]. If this interpretation were supported, however, we would have expected greater integration in these regions to correlate with worse cognitive performance and it would be unlikely that greater integration of the dorsal medial thalamus and red nucleus would be positively correlated with general well-being. Longitudinal studies that can examine the cross-lagged nature of the correlations between changes in brain network integration, change in cognition, and changes in emotion regulation and life satisfaction promise to be most informative about the causal mechanisms associated with individual differences in age-related changes in cognition and well-being.

### 4.2. Association between Network Topology and Performance

We observed that greater local efficiency in the ventral medial prefrontal cortex (VMPFC) was associated with greater processing speed. While these results should be interpreted with care since the outcome would not survive correction for multiple comparisons, we connect this with previous literature and offer an interpretation that can be evaluated in future research. The VMPFC is a primary hub in the DMN and the brain at large [44], [54], and its greater functional connectivity with the posterior cingulate cortex (PCC) in the DMN has been associated with better cognitive performance on composite measures of processing speed, memory, and executive function [13]. In Andrews-Hanna et al. [13] overlapping variance associated with processing speed in composite measures of executive function and memory was not accounted for so it is possible that the general association with cognitive performance would have been attenuated after accounting for processing speed.

Another study that found an association between VMPFC function and processing speed showed that greater task-induced deactivation of the VMPFC following pharmacological up-regulation of norepinephrine and dopamine systems was related to drug-induced improvements in processing speed [55]. Results were interpreted in the context of a theory of adaptive gain control of the locus coeruleus (LC)- norepinephrine (NE) system, where in the service of goal-directed behavior, greater NE augments experience-driven or evoked neuronal responses (excitatory and inhibitory) to modulate their response above ongoing spontaneous activity [56]. The VMPFC receives significant NE innervation from the LC, and it has been shown that stimulation of the LC decreases basal neuronal activity in the VMPFC [57]. Given the VMPFC is highly functionally connected with the PCC in the DMN, making them local neighbors in topological network space (see figure S1), decreased basal activity in the VMPFC may initiate co-deactivation throughout the DMN, and thereby increase the signal-to-noise ratio for neuronal activity involved in coordinating goal-directed behavior. Local efficiency of the VMPFC characterizes the efficacy of co-activation with the PCC rather than its signature close functional distance with many other brain regions that make it a hub; this would be a possible account for why VMPFC local efficiency was more sensitive to an association with processing speed than global efficiency.

### 4.3. Conclusions, Limitations, and Future Directions

This study applied network science methods to measure functional connectivity in the brain, based on mathematical models that have been used for characterizing dynamic network behavior in self-organizing real-world systems, such as social networks or patterns of commercial airplane travel [4], [58]. The study explored the data in regards to aging, performance, and well-being, and demonstrated the richness of the information gained from modeling the brain as a complex network and its potential for generating new insight about brain function.

For future research, it will be important for studies to examine their data using varying spatial scales of network nodes and investigate what resolution of nodes and modules is most sensitive to differences in age and clinical status. Along these lines, it will be critical for future research to examine how relationships between network topology, performance, and well-being change as a function of scale. Examinations of network topology anchored in their comparative clinical relevance promise to complement pure methodological approaches for determining brain network topology.

Related to this future direction, the data used in the current study included three fMRI runs of distinct passive viewing tasks. We tried to control for task-specific global signal changes by regressing out task-related signal from the primary visual cortex, however some task-related effects common across tasks may have still been present in the data. We examined this concern in supplementary power spectral density analysis (see Figures S7 and S8). This analysis demonstrated that while some task-related signal remained in the data, its influence was restricted primarily to the visual cortex in younger and older adults. Since these regions were not prominent in observed age-related differences, we do not feel this residual visual cortex signal significantly influenced the pattern of our results. However, it will be important to assess questions raised here with task-free resting state data to determine the generalizability of our conclusions to a task-independent state. Finally, future studies with a more diverse sample will provide a test for the generalizability of our results beyond the relatively high-functioning older adult group matched to young adults in our analyses.

Overall, the current study used a network science perspective to reveal novel insights about the aging brain and cognition. Network modeling of brain structure and function promises to change the landscape of cognitive neuroscience by adding a toolbox for systems-level analysis of brain function with which to understand the complexities of brain development and behavior across the lifespan. As such this study is a valuable contribution for methodological advancement in application of using network science to address the important societal issue of the aging mind and brain.

## Supporting Information

Figure S1 Local neighbors of the VMPFC for young and old. Voxels in red/orange illustrate where >50% of subjects had at least one direct connection with voxels showing age differences in integration in the VMPFC (blue). Local efficiency of a voxel is a measure of functionally inter-connected direct topological neighbors (excluding that voxel); therefore areas in red/orange are on average the regions included in the local functional network for young and older adults. Young demonstrate intact cohesion of primary DMN regions compared to older adults. Axial slices are Z = 46, 36, 20, −10, −16, −30 shown left to right, in neurological orientation (L = L, R = R).
(TIFF)

Figure S2 Supplemental seed analyses of ROIs for Old>Young. To examine the overlap of the regions showing greater integration for Old>Young with the Salience network, we submitted these ROIs to a seed-based functional connectivity analyses. Seed functional connectivity analyses determine the regions of the brain (voxels) that have the highest correlation with the timeseries of the seed region. The sample used here was an independent sample of 15 older adults (5 males) with 5 minutes of resting state data (TR = 2/TE = 30 ms). On average these adults were 63.9 (SD = 4) years of age, with 15.6 (SD = 3.7) years of education. They were very similar demographically to the current sample and provide an independent comparison sample. Axial slices are the same slices presented in the body of the manuscript for the O>Y comparison. Maps represent Z-statistics, with a height threshold of Z>2.33 and no cluster threshold.
(TIFF)

Figure S3 Conjunction of seeds shown in Figure S2, including orbito-frontal seed and seeds from the current study. Binary conjunction map was created by multiplying individual seed maps from above (each at threshold of Z>2.33), and binarizing the map of common regions. Dorsal anterior cingulate (dACC) region shown overlaps with dACC region in the Salience Network reported in Seeley et al., 2007, and the cerebellar region (Right Crus I) overlaps with the region reported in the Salience Network shown in Habas et al., 2009.
(TIFF)

Figure S4 Preprocessing for functional images, see methods for description of specific algorithms for each preprocessing step (e.g., motion correction, brain extraction, registration) and Network analysis pipeline.
(TIFF)

Figure S5 A mask of common gray matter space across subjects was created by thresholding FSL’s standard space population gray matter tissue prior image (avg152T1_gray.nii) at an intensity value of 50, which represents keeping the top 80% of values. We chose this value based on the histogram of intensity values in the avg152T1_gray.nii image, which indicated that 50 is a conservative minimum for gray matter voxels. Here we illustrate the standard MNI template overlayed with this gray matter mask shown in red.
(TIFF)

Figure S6 Distributions of young and older adult relative motion (a) before and (b) after matching groups for motion. Relative motion refers to the absolute value of the distance between each functional image and the image in the previous time-point within the individual runs. The value per subject represents the average relative motion of the three runs. It was not possible to exactly match on motion. As seen in the figure, some young subjects with more relative motion than older adults were in the analysis. Results suggest this was not enough to create a confound for the Old>Young comparison, there were no significant correlations between average relative motion (mm) and global or local efficiency in the dorsal medial thalamus, red nucleus, or bilateral cerebellum (p>.05 two-tailed).
(TIFF)

Figure S7 Supplemental analysis of influence of task-related signal. We examined the extent to which there was contamination of “spontaneous” signal fluctuation by task signal, following a nuisance regression that included signal from the primary visual cortex. First, we present the average power spectrums for the fully preprocessed data (i.e., data that entered the graph theoretical analyses), for (a) younger and (b) older adults. Note the data were bandpass filtered to.008< f <.08 Hz. The task signal would be strongest at approximately.02 to.03 Hz. Note across the entire brain, averaged across participants, there is a “bump” in our data spectrums that is not evident in the exemplar resting state data (c) from a sample of demographically similar older adults. This suggests task-related signal remained in the data. The gray shaded regions reflect ±1 SE from the group mean.
(TIFF)

Figure S8 Regional distribution of signal left in the data after nuisance regression, as determined by a spatial power spectral density analyses. This analysis was done with the fsl tool *fslpspec*, and computes the voxel-wise power spectrum below the Nyquist limit (1/(2*TR)). To summarize the results of this analysis, we present the average power for two frequency bins, including (1) 0–016 Hz (first ‘bump’ in Figure S7) and (2) .019−.03 Hz (second ‘bump’ in Figure S8). This analysis demonstrates that while task-related signal remained in the data, its influence was primarily restricted to the primary visual cortex in younger and older adults.
(TIFF)

Figure S9 Stability of S threshold. (a) Stability of the S threshold was examined for S = 2.5 and S = 3.0. The y axis represents final average S = log(N)/log(K) for each subject’s network. The first 30 subjects are older adults, followed by the younger adults. Stability of S was somewhat greater for S = 2.5. (b) Mean path length across all voxels for S = 2.5 was 5.08 (SD = .36) and mean path length for S = 3.0 was 6.39 (SD = .45). Individual path lengths for both thresholds are visualized below. The first 30 subjects are older adults, followed by the younger adults.
(TIFF)

Table S1 Multiple linear regressions predicting single task reaction time from global and local efficiency in the default mode network.
(DOCX)

Table S2 Multiple linear regressions predicting single task reaction time from global and local efficiency in the fronto-parietal network.
(DOCX)

Table S3 Multiple linear regressions predicting single task reaction time from global and local efficiency in the cerebellar and sub-cortical network.
(DOCX)

Table S4 Multiple linear regressions predicting set-switching reaction time from global and local efficiency in the default mode network.
(DOCX)

Table S5 Multiple linear regressions predicting set-switching reaction time from global and local efficiency in the fronto-parietal network.
(DOCX)

Table S6 Multiple linear regressions predicting set-switching reaction time from global and local efficiency in the cerebellar and sub-cortical network.
(DOCX)

Table S7 Multiple linear regressions predicting SPWM reaction time from global and local efficiency in the default mode network.
(DOCX)

Table S8 Multiple linear regressions predicting SPWM reaction time from global and local efficiency in the fronto-parietal network.
(DOCX)

Table S9 Multiple linear regressions predicting SPWM reaction time from global and local efficiency in the cerebellar and sub-cortical network.
(DOCX)

Table S10 Multiple linear regressions predicting SPWM accuracy from global and local efficiency in the default mode network.
(DOCX)

Table S11 Multiple linear regressions predicting SPWM accuracy from global and local efficiency in the fronto-parietal network.
(DOCX)

Table S12 Multiple linear regressions predicting SPWM accuracy from global and local efficiency in the cerebellar and sub-cortical network.
(DOCX)
