# Supplementary figures and images for: Aging Brain from a Network Science Perspective: Something to Be Positive About?
Source: PLoS One. 2013 Nov 6;8(11):e78345. doi: 10.1371/journal.pone.0078345 (PMC3819386; doi:10.1371/journal.pone.0078345)

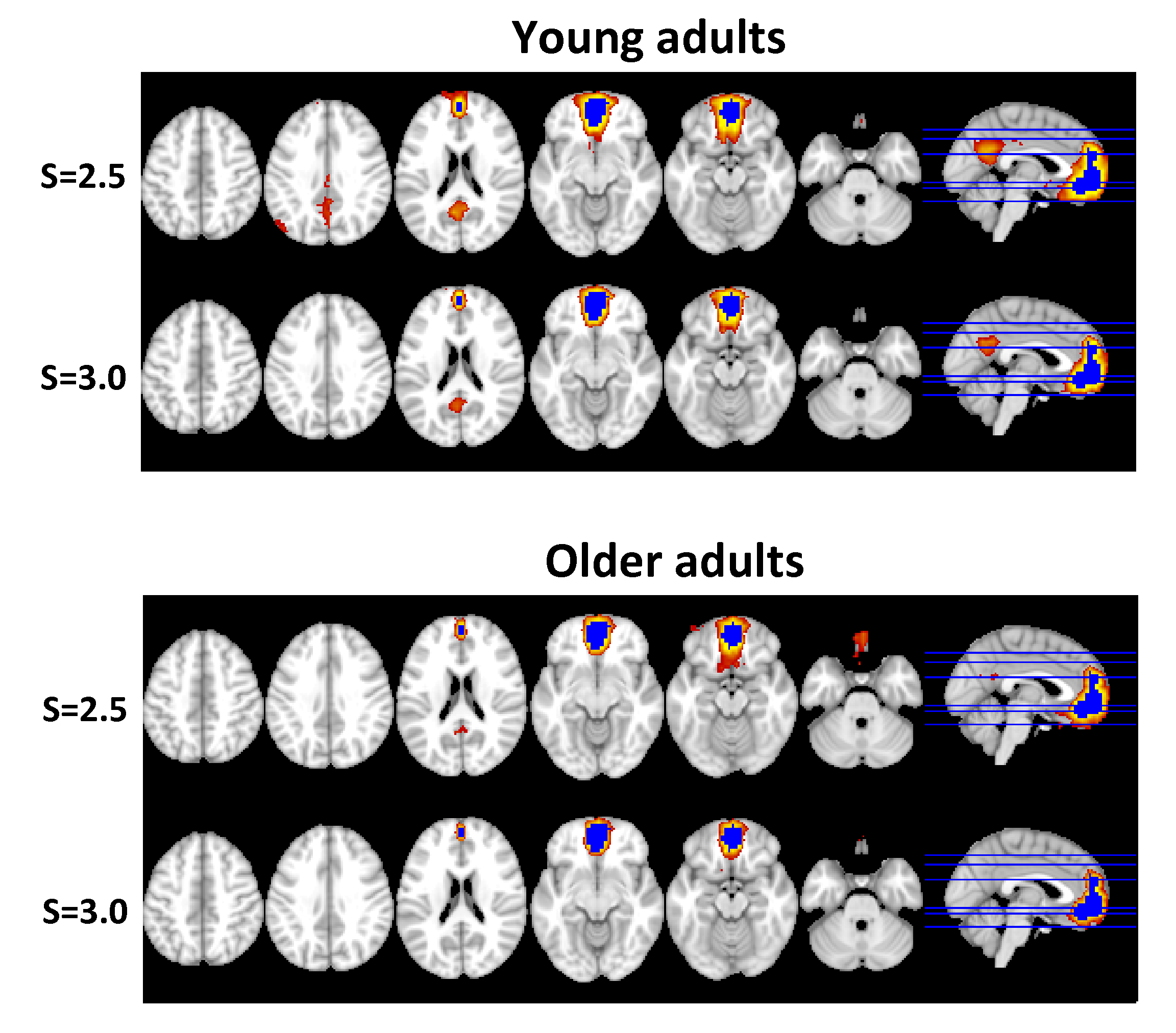

Supplement: Figure S1 — Local neighbors of the VMPFC for young and old. Voxels in red/orange illustrate where >50% of subjects had at least one direct connection with voxels showing age differences in integration in the VMPFC (blue). Local efficiency of a voxel is a measure of functionally inter-connected direct topological neighbors (excluding that voxel); therefore areas in red/orange are on average the regions included in the local functional network for young and older adults. Young demonstrate intact cohesion of primary DMN regions compared to older adults. Axial slices are Z = 46, 36, 20, −10, −16, −30 shown left to right, in neurological orientation (L = L, R = R). (TIFF) [file pone.0078345.s001.tiff]

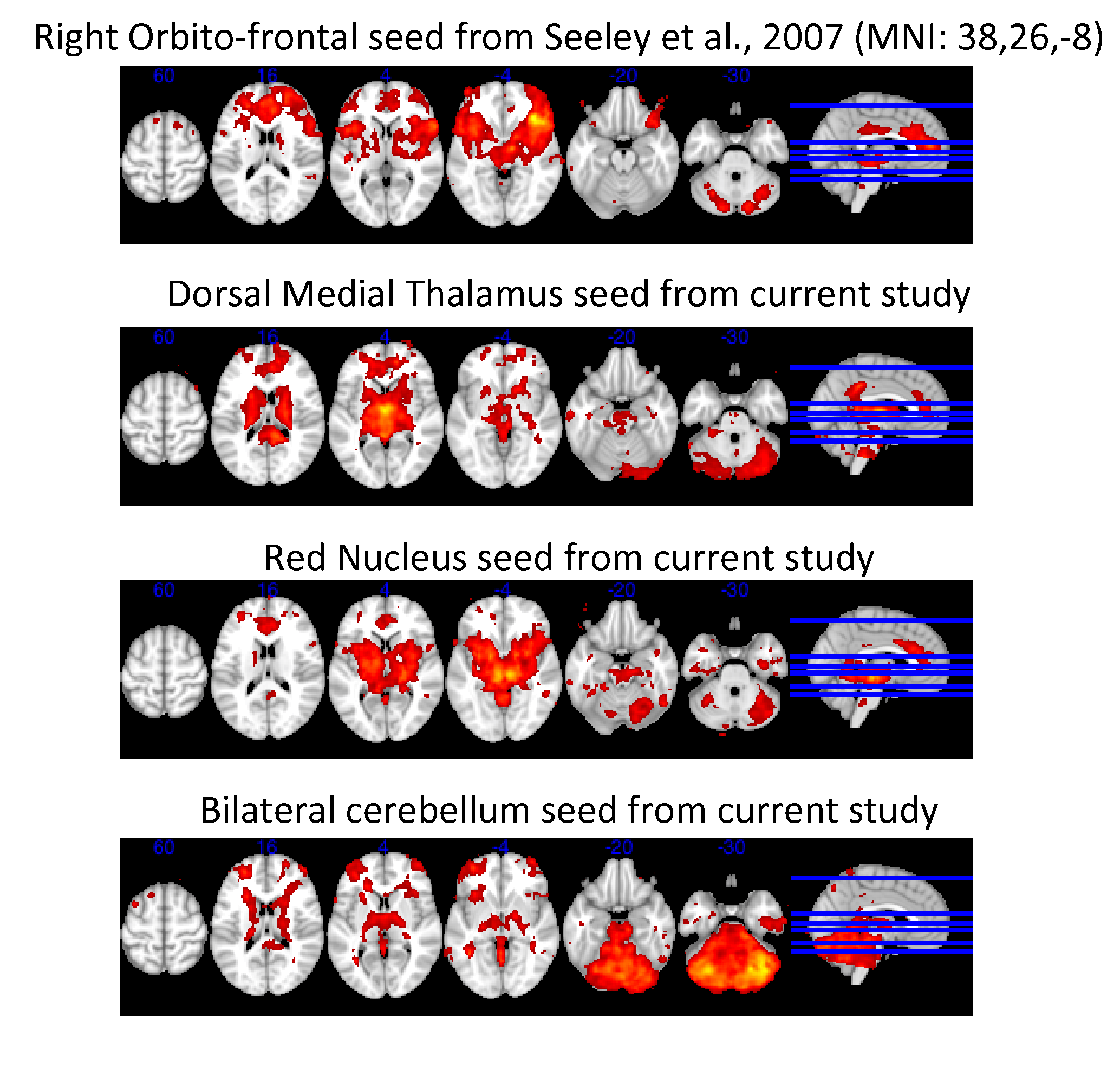

Supplement: Figure S2 — Supplemental seed analyses of ROIs for Old>Young. To examine the overlap of the regions showing greater integration for Old>Young with the Salience network, we submitted these ROIs to a seed-based functional connectivity analyses. Seed functional connectivity analyses determine the regions of the brain (voxels) that have the highest correlation with the timeseries of the seed region. The sample used here was an independent sample of 15 older adults (5 males) with 5 minutes of resting state data (TR = 2/TE = 30 ms). On average these adults were 63.9 (SD = 4) years of age, with 15.6 (SD = 3.7) years of education. They were very similar demographically to the current sample and provide an independent comparison sample. Axial slices are the same slices presented in the body of the manuscript for the O>Y comparison. Maps represent Z-statistics, with a height threshold of Z>2.33 and no cluster threshold. (TIFF) [file pone.0078345.s002.tiff]

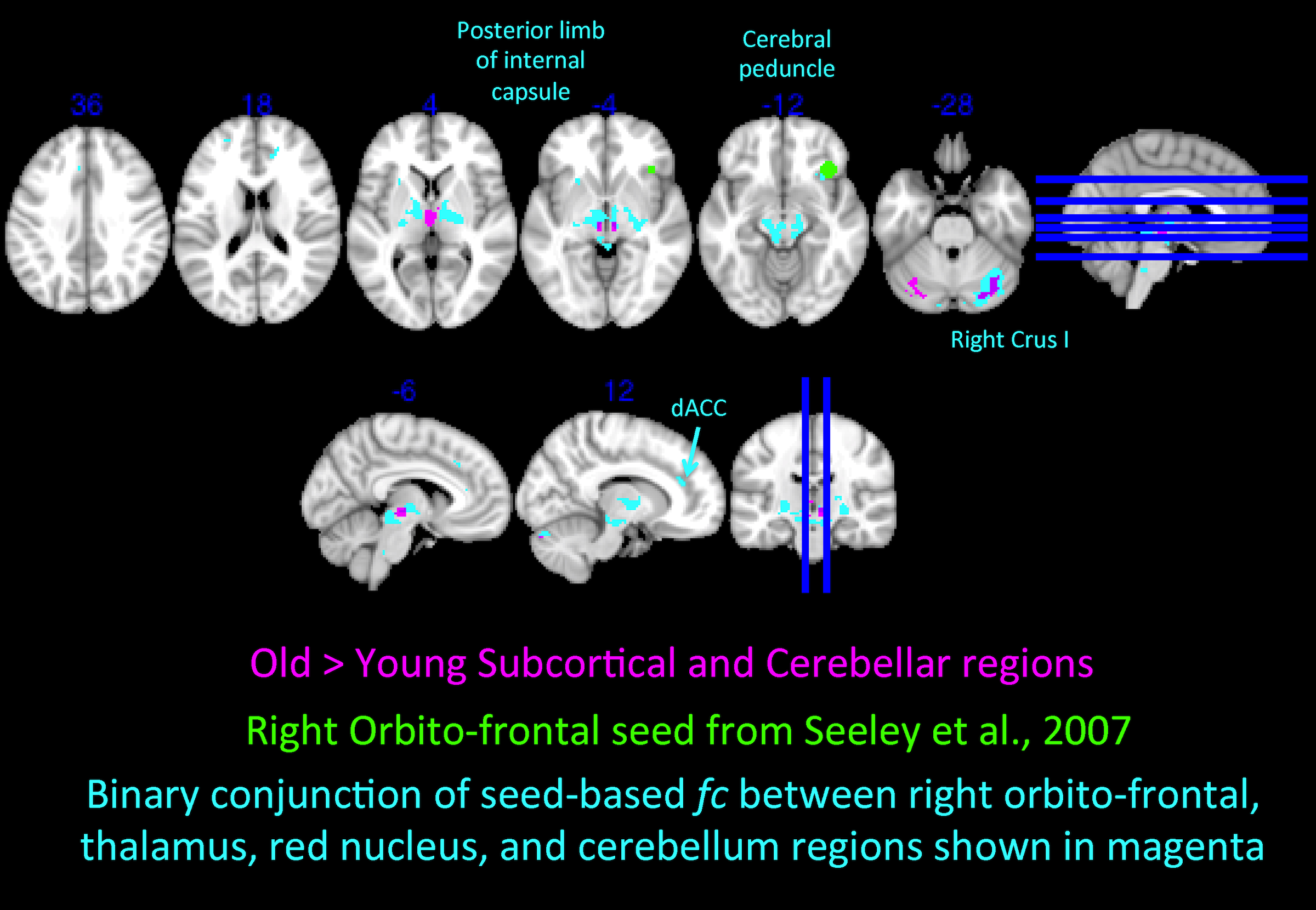

Supplement: Figure S3 — Conjunction of seeds shown in Figure S2, including orbito-frontal seed and seeds from the current study. Binary conjunction map was created by multiplying individual seed maps from above (each at threshold of Z>2.33), and binarizing the map of common regions. Dorsal anterior cingulate (dACC) region shown overlaps with dACC region in the Salience Network reported in Seeley et al., 2007, and the cerebellar region (Right Crus I) overlaps with the region reported in the Salience Network shown in Habas et al., 2009. (TIFF) [file pone.0078345.s003.tiff]

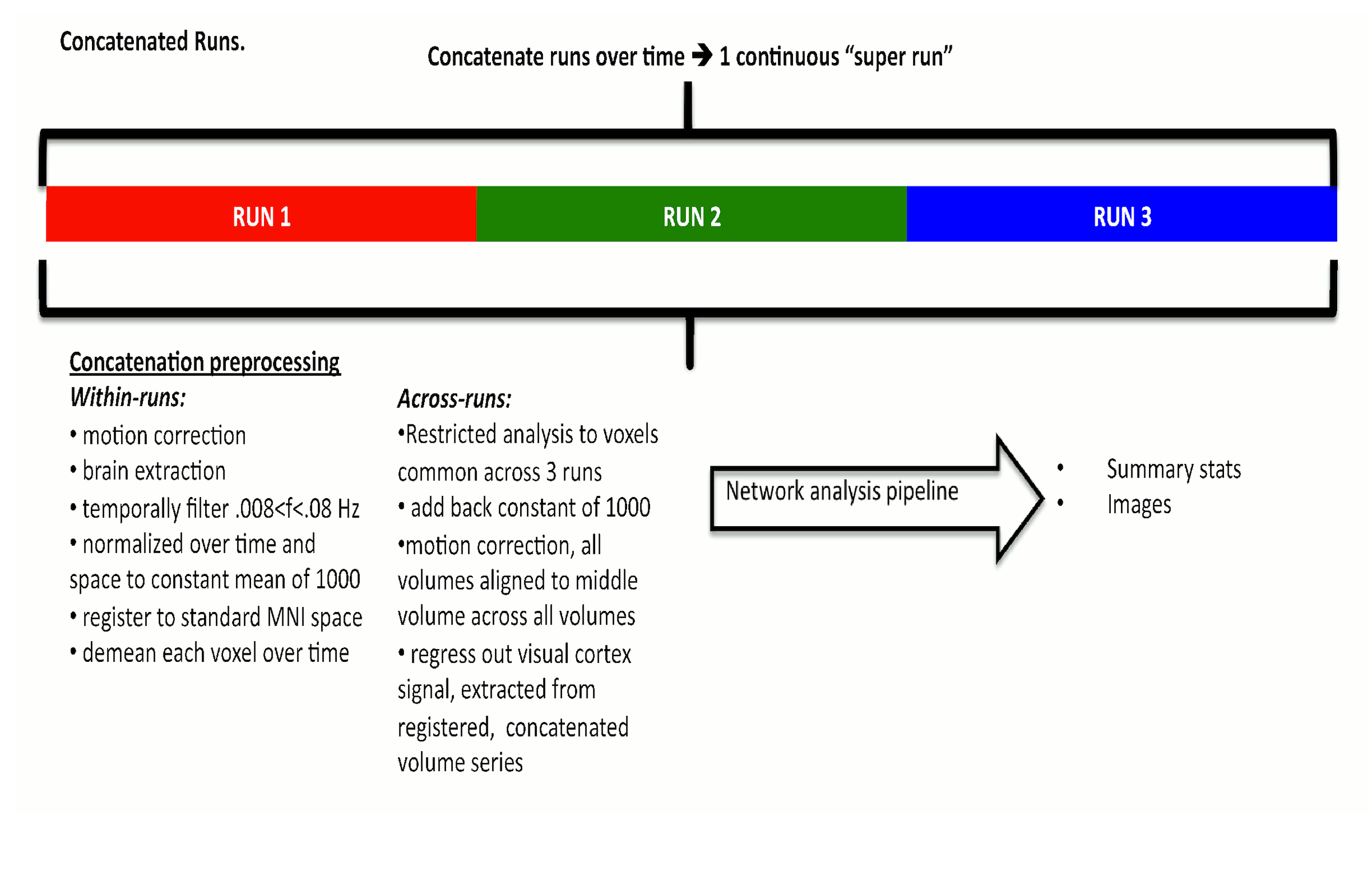

Supplement: Figure S4 — Preprocessing for functional images, see methods for description of specific algorithms for each preprocessing step (e.g., motion correction, brain extraction, registration) and Network analysis pipeline. (TIFF) [file pone.0078345.s004.tiff]

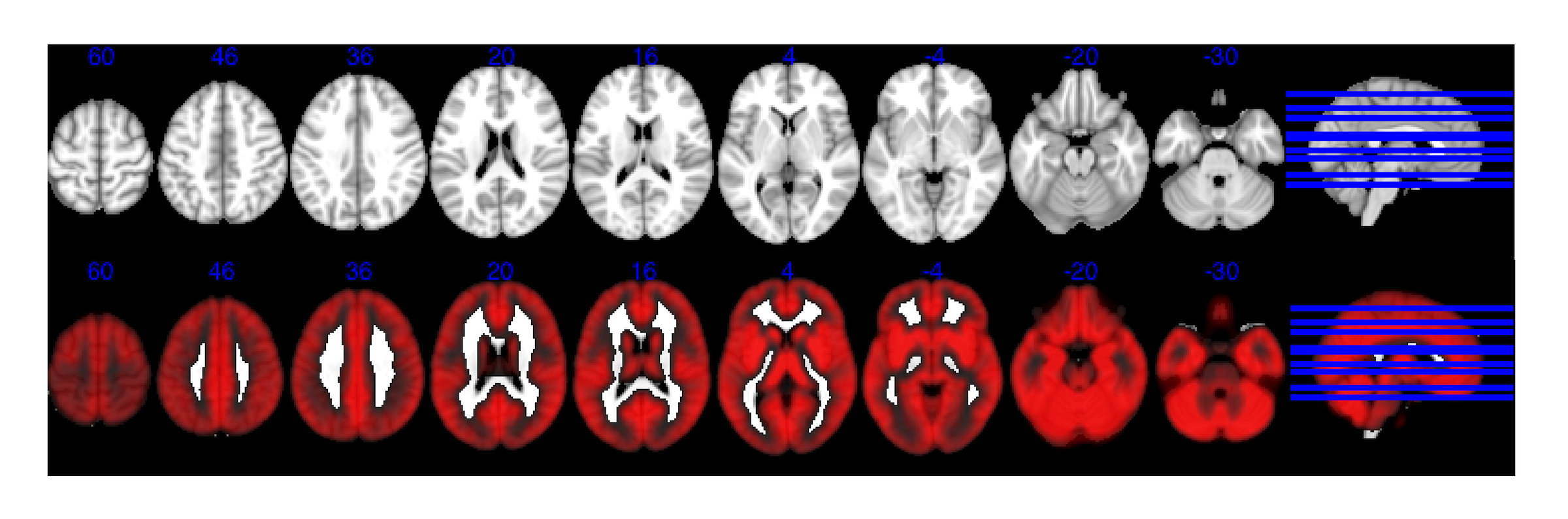

Supplement: Figure S5 — A mask of common gray matter space across subjects was created by thresholding FSL’s standard space population gray matter tissue prior image (avg152T1_gray.nii) at an intensity value of 50, which represents keeping the top 80% of values. We chose this value based on the histogram of intensity values in the avg152T1_gray.nii image, which indicated that 50 is a conservative minimum for gray matter voxels. Here we illustrate the standard MNI template overlayed with this gray matter mask shown in red. (TIFF) [file pone.0078345.s005.tiff]

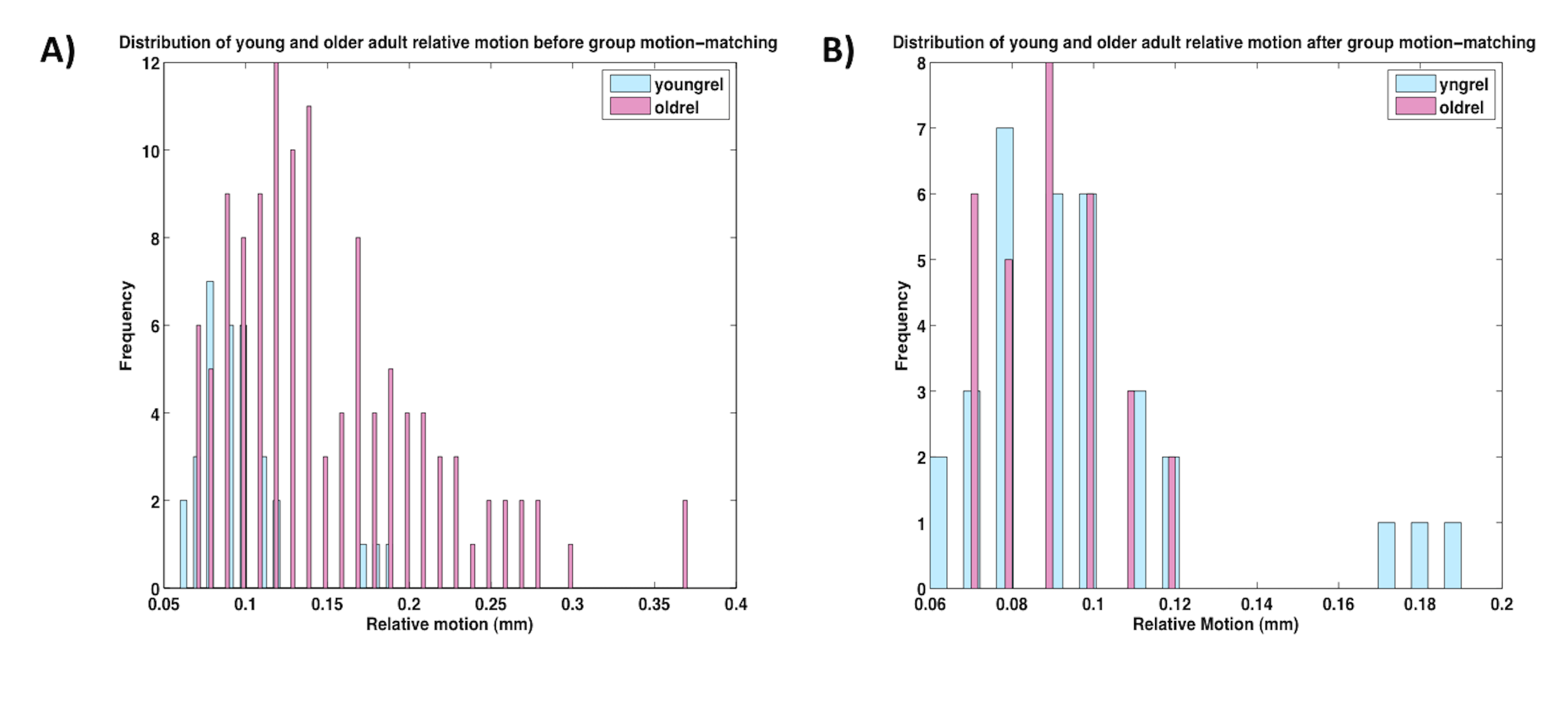

Supplement: Figure S6 — Distributions of young and older adult relative motion (a) before and (b) after matching groups for motion. Relative motion refers to the absolute value of the distance between each functional image and the image in the previous time-point within the individual runs. The value per subject represents the average relative motion of the three runs. It was not possible to exactly match on motion. As seen in the figure, some young subjects with more relative motion than older adults were in the analysis. Results suggest this was not enough to create a confound for the Old>Young comparison, there were no significant correlations between average relative motion (mm) and global or local efficiency in the dorsal medial thalamus, red nucleus, or bilateral cerebellum (p>.05 two-tailed). (TIFF) [file pone.0078345.s006.tiff]

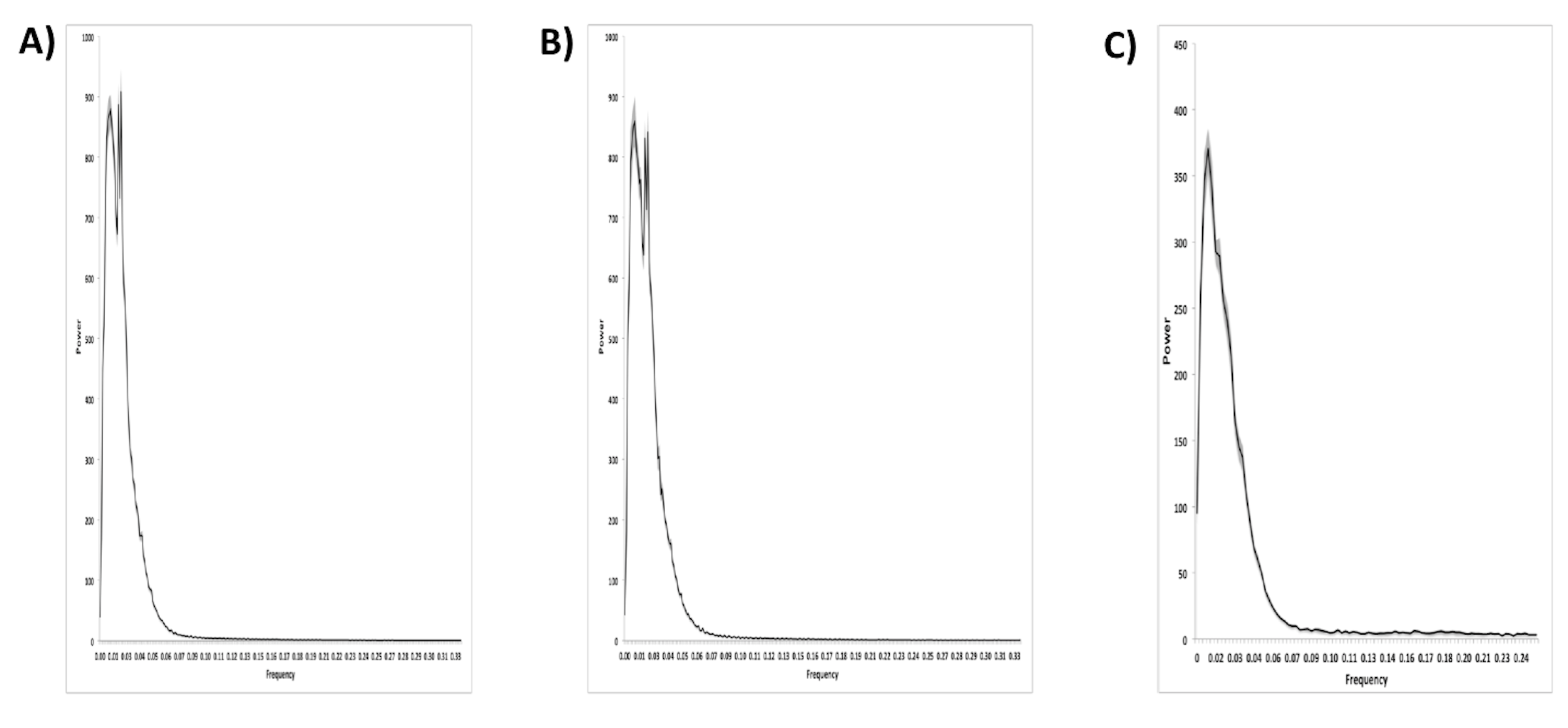

Supplement: Figure S7 — Supplemental analysis of influence of task-related signal. We examined the extent to which there was contamination of “spontaneous” signal fluctuation by task signal, following a nuisance regression that included signal from the primary visual cortex. First, we present the average power spectrums for the fully preprocessed data (i.e., data that entered the graph theoretical analyses), for (a) younger and (b) older adults. Note the data were bandpass filtered to.008< f <.08 Hz. The task signal would be strongest at approximately.02 to.03 Hz. Note across the entire brain, averaged across participants, there is a “bump” in our data spectrums that is not evident in the exemplar resting state data (c) from a sample of demographically similar older adults. This suggests task-related signal remained in the data. The gray shaded regions reflect ±1 SE from the group mean. (TIFF) [file pone.0078345.s007.tiff]

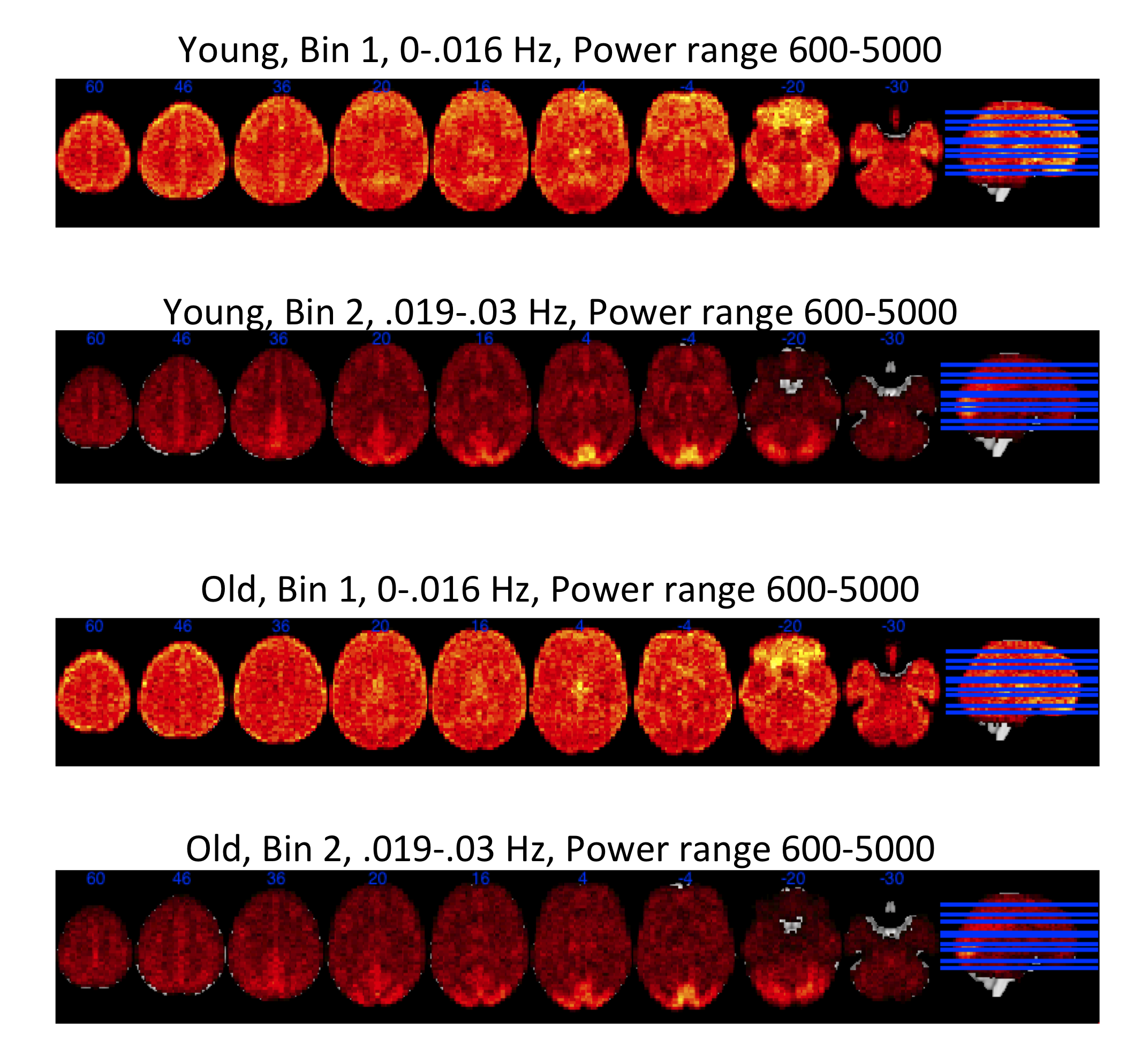

Supplement: Figure S8 — Regional distribution of signal left in the data after nuisance regression, as determined by a spatial power spectral density analyses. This analysis was done with the fsl tool fslpspec, and computes the voxel-wise power spectrum below the Nyquist limit (1/(2*TR)). To summarize the results of this analysis, we present the average power for two frequency bins, including (1) 0–016 Hz (first ‘bump’ in Figure S7) and (2) .019−.03 Hz (second ‘bump’ in Figure S8). This analysis demonstrates that while task-related signal remained in the data, its influence was primarily restricted to the primary visual cortex in younger and older adults. (TIFF) [file pone.0078345.s008.tiff]

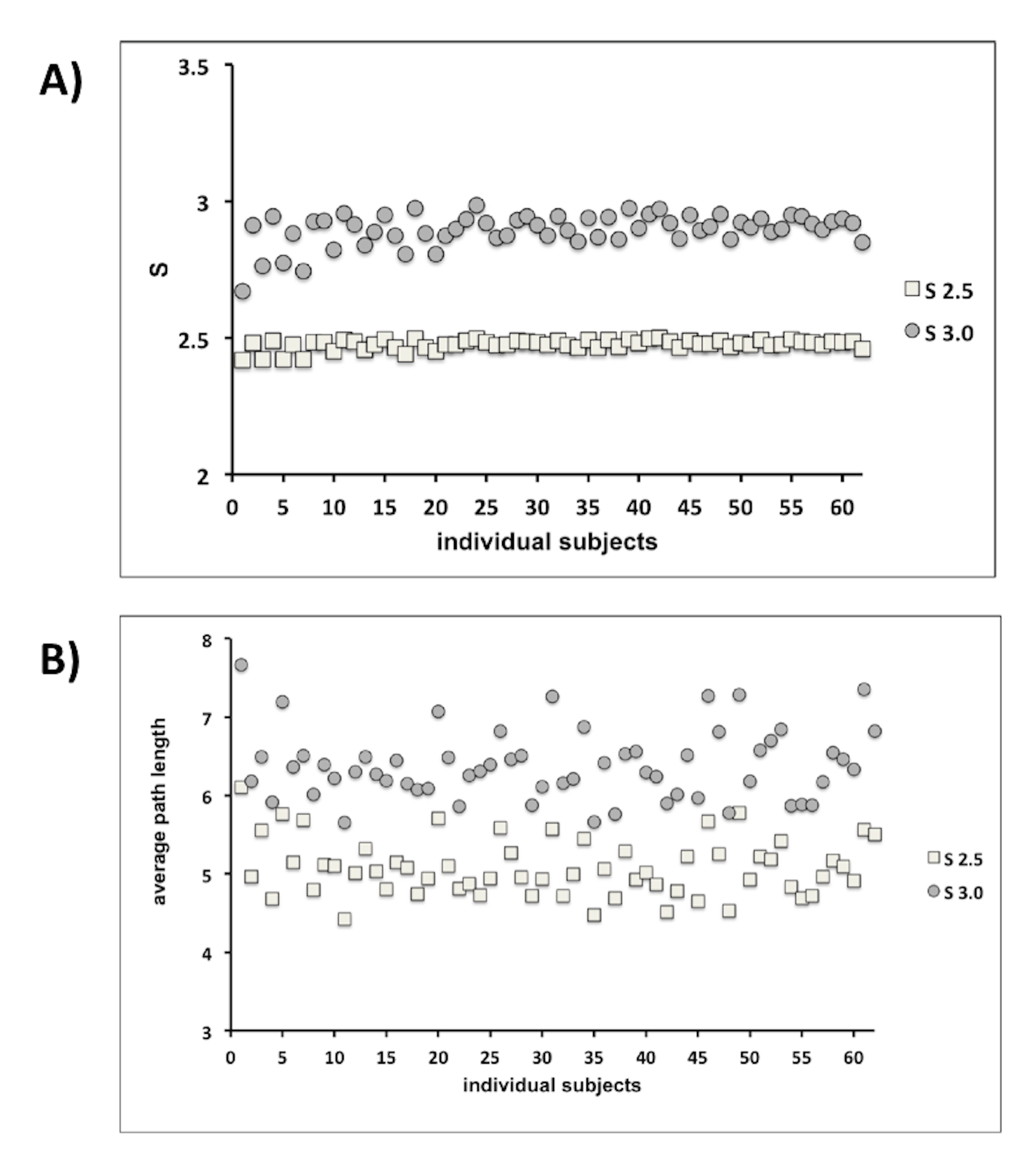

Supplement: Figure S9 — Stability of S threshold. (a) Stability of the S threshold was examined for S = 2.5 and S = 3.0. The y axis represents final average S = log(N)/log(K) for each subject’s network. The first 30 subjects are older adults, followed by the younger adults. Stability of S was somewhat greater for S = 2.5. (b) Mean path length across all voxels for S = 2.5 was 5.08 (SD = .36) and mean path length for S = 3.0 was 6.39 (SD = .45). Individual path lengths for both thresholds are visualized below. The first 30 subjects are older adults, followed by the younger adults. (TIFF) [file pone.0078345.s009.tiff]
