# Supplementary material for: Aging Brain from a Network Science Perspective: Something to Be Positive About?
Source: PLoS One. 2013 Nov 6;8(11):e78345. doi: 10.1371/journal.pone.0078345 (PMC3819386; doi:10.1371/journal.pone.0078345)
Supplement: Table S1 — Multiple linear regressions predicting single task reaction time from global and local efficiency in the default mode network. (DOCX) [file pone.0078345.s010.docx]

**Table S1**

| ROIs from the **Default Mode Network**  DV: **Single task reaction time (processing speed)** | | | | | | | | | |
| --- | --- | --- | --- | --- | --- | --- | --- | --- | --- |
|  |  | Global Efficiency | | | | Local Efficiency | | | |
|  |  | 250 | | 300 | | 250 | | 300 | |
|  |  | β | R^2^ | β | R^2^ | β | R^2^ | β | R^2^ |
| Step 1 |  |  | .40 |  | .40 |  | .40 |  | .40 |
|  | Age | -.62*** |  | -.62*** |  | -.62*** |  | -.62*** |  |
|  | Sex | -.06 |  | -.06 |  | -.06 |  | -.06 |  |
|  |  |  |  |  |  |  |  |  |  |
| Step 2 | PCC |  | .42 |  | .42 |  | .41 |  | .41 |
|  | Age | -.54*** |  | -.53*** |  | -.61*** |  | -.58*** |  |
|  | Sex | -.08 |  | -.08 |  | -.07 |  | -.08 |  |
|  | ROI | -.17 |  | -.16 |  | -.14 |  | -.19 |  |
|  | Age x ROI | -.02 |  | -.04 |  | .11 |  | .12 |  |
| Step 2 | AntPCC |  | .41 |  | .41 |  | .46^†^ |  | .42 |
|  | Age | -.59*** |  | -.58*** |  | -.63*** |  | -.58*** |  |
|  | Sex | -.05 |  | -.05 |  | -.06 |  | -.05 |  |
|  | ROI | .04 |  | .07 |  | .29* |  | .09 |  |
|  | Age x ROI | -.14 |  | -.15 |  | -.32* |  | -.20 |  |
| Step 2 | ParOcc |  | .41 |  | .41 |  | .40 |  | .41 |
|  | Age | -.56*** |  | -.57*** |  | -.66*** |  | -.64*** |  |
|  | Sex | -.07 |  | -.07 |  | -.08 |  | -.08 |  |
|  | ROI | -.16 |  | -.15 |  | -.01 |  | -.08 |  |
|  | Age x ROI | .06 |  | .06 |  | .10 |  | .15 |  |
| Step 2 | VMPFC |  | .45^†^ |  | .44 |  | .44 |  | .45^†^ |
|  | Age | -.49*** |  | -.49*** |  | -.49** |  | -.43** |  |
|  | Sex | -.07 |  | -.08 |  | -.10 |  | -.10 |  |
|  | ROI | -.28* |  | -.26^†^ |  | -.39* |  | -.39* |  |
|  | Age x ROI | .03 |  | .01 |  | .24 |  | .13 |  |

β p-value: ^†^p<.10, *p<.05, **p<.01, ***p<.001; R^2^ p-value symbol represents statistical significance of R Square change.
