# Supplementary material for: Aging Brain from a Network Science Perspective: Something to Be Positive About?
Source: PLoS One. 2013 Nov 6;8(11):e78345. doi: 10.1371/journal.pone.0078345 (PMC3819386; doi:10.1371/journal.pone.0078345)
Supplement: Table S2 — Multiple linear regressions predicting single task reaction time from global and local efficiency in the fronto-parietal network. (DOCX) [file pone.0078345.s011.docx]

**Table S2**

| ROIs from the **Fronto-parietal Network**  DV: **Single task reaction time (processing speed)** | | | | | | | | | |
| --- | --- | --- | --- | --- | --- | --- | --- | --- | --- |
|  |  | Global Efficiency | | | | Local Efficiency | | | |
|  |  | 250 | | 300 | | 250 | | 300 | |
|  |  | β | R^2^ | β | R^2^ | β | R^2^ | β | R^2^ |
| Step 1 |  |  | .40 |  | .40 |  | .40 |  | .40 |
|  | Age | -.62*** |  | -.62*** |  | -.62*** |  | -.62*** |  |
|  | Sex | -.06 |  | -.06 |  | -.06 |  | -.06 |  |
|  |  |  |  |  |  |  |  |  |  |
| Step 2 | SupPar |  | .41 |  | .41 |  | .42 |  | .40 |
|  | Age | -.56*** |  | -.56*** |  | -.56*** |  | -.60*** |  |
|  | Sex | -.08 |  | -.08 |  | -.06 |  | -.07 |  |
|  | ROI | -.08 |  | -.06 |  | .03 |  | .03 |  |
|  | Age x ROI | -.08 |  | -.08 |  | -.19 |  | -.10 |  |
| Step 2 | LatOcc |  | .45^†^ |  | .43 |  | .42 |  | .46^†^ |
|  | Age | -.52*** |  | -.52*** |  | -.57*** |  | -.61*** |  |
|  | Sex | -.04 |  | -.06 |  | -.06 |  | -.07 |  |
|  | ROI | -.26^†^ |  | -.17 |  | -.20 |  | -.28* |  |
|  | Age x ROI | .02 |  | -.06 |  | .09 |  | .29* |  |
| Step 2 | LingFus |  | .44 |  | .45^†^ |  | .40 |  | .41 |
|  | Age | -.50*** |  | -.48*** |  | -.58*** |  | -.68*** |  |
|  | Sex | -.06 |  | -.06 |  | -.06 |  | -.09 |  |
|  | ROI | -.20 |  | -.23^†^ |  | -.06 |  | .01 |  |
|  | Age x ROI | -.06 |  | -.07 |  | -.01 |  | .14 |  |

β p-value: ^†^p<.10, *p<.05, **p<.01, ***p<.001; R^2^ p-value symbol represents statistical significance of R Square change.
