# Supplementary material for: Aging Brain from a Network Science Perspective: Something to Be Positive About?
Source: PLoS One. 2013 Nov 6;8(11):e78345. doi: 10.1371/journal.pone.0078345 (PMC3819386; doi:10.1371/journal.pone.0078345)
Supplement: Table S3 — Multiple linear regressions predicting single task reaction time from global and local efficiency in the cerebellar and sub-cortical network. (DOCX) [file pone.0078345.s012.docx]

**Table S3**

| ROIs in **Cerebellum and Subcortical structures**  DV: **Single task reaction time (processing speed)** | | | | | | | | | |
| --- | --- | --- | --- | --- | --- | --- | --- | --- | --- |
|  |  | Global Efficiency | | | | Local Efficiency | | | |
|  |  | 250 | | 300 | | 250 | | 300 | |
|  |  | β | R^2^ | β | R^2^ | β | R^2^ | β | R^2^ |
| Step 1 |  |  | .40 |  | .40 |  | .40 |  | .40 |
|  | Age | -.62*** |  | -.62*** |  | -.62*** |  | -.62*** |  |
|  | Sex | -.06 |  | -.06 |  | -.06 |  | -.06 |  |
|  |  |  |  |  |  |  |  |  |  |
| Step 2 | CBM |  | .41 |  | .42 |  | .45 |  | .43 |
|  | Age | -.55*** |  | -.54*** |  | -.57*** |  | -.62*** |  |
|  | Sex | -.04 |  | -.05 |  | -.01 |  | -.07 |  |
|  | ROI | .07 |  | .08 |  | .24* |  | .16 |  |
|  | Age x ROI | -.14 |  | -.16 |  | .01 |  | .16 |  |
| Step 2 | RedNuc |  | .42 |  | .42 |  | .42 |  | .42 |
|  | Age | -.51*** |  | -.51*** |  | -.56*** |  | -.64*** |  |
|  | Sex | -.07 |  | -.08 |  | -.07 |  | -.10 |  |
|  | ROI | .12 |  | .11 |  | .17 |  | .10 |  |
|  | Age x ROI | -.14 |  | -.17 |  | .03 |  | .16 |  |
| Step 2 | DMThal |  | .41 |  | .42 |  | .41 |  | .44 |
|  | Age | -.58*** |  | -.56*** |  | -.64*** |  | -.70*** |  |
|  | Sex | -.06 |  | -.07 |  | -.05 |  | -.07 |  |
|  | ROI | -.02 |  | -.01 |  | .10 |  | .16 |  |
|  | Age x ROI | -.12 |  | -.15 |  | -.01 |  | .16 |  |

β p-value: ^†^p<.10, *p<.05, **p<.01, ***p<.001; R^2^ p-value symbol represents statistical significance of R Square change.
