# Supplementary material for: Aging Brain from a Network Science Perspective: Something to Be Positive About?
Source: PLoS One. 2013 Nov 6;8(11):e78345. doi: 10.1371/journal.pone.0078345 (PMC3819386; doi:10.1371/journal.pone.0078345)
Supplement: Table S5 — Multiple linear regressions predicting set-switching reaction time from global and local efficiency in the fronto-parietal network. (DOCX) [file pone.0078345.s014.docx]

**Table S5**

| ROIs from the **Fronto-parietal Network**  DV: **Switch RT (set-switching speed)** | | | | | | | | | |
| --- | --- | --- | --- | --- | --- | --- | --- | --- | --- |
|  |  | Global Efficiency | | | | Local Efficiency | | | |
|  |  | 250 | | 300 | | 250 | | 300 | |
|  |  | β | R^2^ | β | R^2^ | β | R^2^ | β | R^2^ |
| Step 1 |  |  | .62 |  | .62 |  |  |  | .62 |
|  | Age | -.31** |  | -.31** |  | -.31** |  | -.31** |  |
|  | Sex | .13 |  | .13 |  | .13 |  | .13 |  |
|  | Single RT | .56*** |  | .56*** |  | .56*** |  | .56*** |  |
|  |  |  |  |  |  |  |  |  |  |
| Step 2 | SupPar |  | .62 |  | .63 |  | .62 |  | .62 |
|  | Age | -.33** |  | -.34** |  | -.36 |  | -.32** |  |
|  | Sex | .14 |  | .14 |  | .15^†^ |  | .13 |  |
|  | Single RT | .57*** |  | .57*** |  | .56*** |  | .56** |  |
|  | ROI | .12 |  | .15 |  | .13 |  | .01 |  |
|  | Age x ROI | -.07 |  | -.10 |  | -.05 |  | .04 |  |
| Step 2 | LatOcc |  | .62 |  | .62 |  | .62 |  | .62 |
|  | Age | -.31** |  | -.32** |  | -.32** |  | -.33** |  |
|  | Sex | .13 |  | .13 |  | .12 |  | .12 |  |
|  | Single RT | .57*** |  | .57*** |  | .54*** |  | .56*** |  |
|  | ROI | .02 |  | .04 |  | -.09 |  | .02 |  |
|  | Age x ROI | -.01 |  | -.02 |  | .11 |  | .03 |  |
| Step 2 | LingFus |  | .62 |  | .62 |  | .64 |  | .63 |
|  | Age | -.30* |  | -.30* |  | -.25* |  | -.28* |  |
|  | Sex | .13 |  | .13 |  | .13 |  | .12 |  |
|  | Single RT | .55*** |  | .55*** |  | .55*** |  | .55*** |  |
|  | ROI | -.05 |  | -.06 |  | -.17^†^ |  | -.14 |  |
|  | Age x ROI | .03 |  | .02 |  | .08 |  | .08 |  |

β p-value: ^†^p<.10, *p<.05, **p<.01, ***p<.001; R^2^ p-value symbol represents statistical significance of R Square change.
