# Supplementary material for: Aging Brain from a Network Science Perspective: Something to Be Positive About?
Source: PLoS One. 2013 Nov 6;8(11):e78345. doi: 10.1371/journal.pone.0078345 (PMC3819386; doi:10.1371/journal.pone.0078345)
Supplement: Table S6 — Multiple linear regressions predicting set-switching reaction time from global and local efficiency in the cerebellar and sub-cortical network. (DOCX) [file pone.0078345.s015.docx]

**Table S6**

| ROIs in **Cerebellum and Subcortical structures**  DV: **Switch RT (set-switching speed)** | | | | | | | | | |
| --- | --- | --- | --- | --- | --- | --- | --- | --- | --- |
|  |  | Global Efficiency | | | | Local Efficiency | | | |
|  |  | 250 | | 300 | | 250 | | 300 | |
|  |  | β | R^2^ | β | R^2^ | β | R^2^ | β | R^2^ |
| Step 1 |  |  | .62 |  | .62 |  | .62 |  | .62 |
|  | Age | -.31** |  | -.31** |  | -.31** |  | -.31** |  |
|  | Sex | .13 |  | .13 |  | .13 |  | .13 |  |
|  | Single RT | .56*** |  | .56*** |  | .56*** |  | .56*** |  |
|  |  |  |  |  |  |  |  |  |  |
| Step 2 | CBM |  | .62 |  | .62 |  | .63 |  | .62 |
|  | Age | -.30* |  | -.30^†^ |  | -.33** |  | -.33** |  |
|  | Sex | .15 |  | .15^†^ |  | .15^†^ |  | .12 |  |
|  | Single RT | .55*** |  | .55*** |  | .52*** |  | .55*** |  |
|  | ROI | .07 |  | .07 |  | .13 |  | .02 |  |
|  | Age x ROI | -.01 |  | -.01 |  | .07 |  | .05 |  |
| Step 2 | RedNuc |  | .62 |  | .63 |  | .63 |  | .62 |
|  | Age | -.35** |  | -.36** |  | -.35** |  | -.35** |  |
|  | Sex | .14 |  | .15^†^ |  | .13 |  | .13 |  |
|  | Single RT | .58*** |  | .58*** |  | .58*** |  | .56*** |  |
|  | ROI | -.10 |  | -.12 |  | -.10 |  | -.09 |  |
|  | Age x ROI | .02 |  | .03 |  | .03 |  | .03 |  |
| Step 2 | DMThal |  | .62 |  | .62 |  | .62 |  | .62 |
|  | Age | -.27* |  | -.27* |  | -.33** |  | -.33** |  |
|  | Sex | .14 |  | .14 |  | .12 |  | .12 |  |
|  | Single RT | .56*** |  | .56*** |  | .56*** |  | .55*** |  |
|  | ROI | .09 |  | .09 |  | .01 |  | .01 |  |
|  | Age x ROI | -.01 |  | -.02 |  | .06 |  | .05 |  |

β p-value: ^†^p<.10, *p<.05, **p<.01, ***p<.001; R^2^ p-value symbol represents statistical significance of R Square change.
