# Supplementary material for: Aging Brain from a Network Science Perspective: Something to Be Positive About?
Source: PLoS One. 2013 Nov 6;8(11):e78345. doi: 10.1371/journal.pone.0078345 (PMC3819386; doi:10.1371/journal.pone.0078345)
Supplement: Table S9 — Multiple linear regressions predicting SPWM reaction time from global and local efficiency in the cerebellar and sub-cortical network. (DOCX) [file pone.0078345.s018.docx]

**Table S9**

| ROIs in **Cerebellum and Subcortical structures**  DV: **SPWM RT (spatial working memory speed)** | | | | | | | | | |
| --- | --- | --- | --- | --- | --- | --- | --- | --- | --- |
|  |  | Global Efficiency | | | | Local Efficiency | | | |
|  |  | 250 | | 300 | | 250 | | 300 | |
|  |  | β | R^2^ | β | R^2^ | β | R^2^ | β | R^2^ |
| Step 1 |  |  | .62 |  | .62 |  | .62 |  | .62 |
|  | Age | -.25* |  | -.25* |  | -.25* |  | -.25* |  |
|  | Sex | .13 |  | .13 |  | .13 |  | .13 |  |
|  | Single RT | .61** |  | .61** |  | .61** |  | .61** |  |
|  |  |  |  |  |  |  |  |  |  |
| Step 2 | CBM |  | .63 |  | .63 |  | .63 |  | .64 |
|  | Age | -.26* |  | -.26* |  | -.22^†^ |  | -.19 |  |
|  | Sex | .15 |  | .15^†^ |  | .13 |  | .16^†^ |  |
|  | Single RT | .63*** |  | .63*** |  | .63*** |  | .65*** |  |
|  | ROI | .04 |  | .05 |  | -.06 |  | -.06 |  |
|  | Age x ROI | .07 |  | .10 |  | -.09 |  | -.16 |  |
| Step 2 | RedNuc |  | .63 |  | .64 |  | .63 |  | .67* |
|  | Age | -.23^†^ |  | -.24^†^ |  | -.23^†^ |  | -.23* |  |
|  | Sex | .13 |  | .13 |  | .15^†^ |  | .18* |  |
|  | Single RT | .62*** |  | .63*** |  | .62*** |  | .66*** |  |
|  | ROI | .08 |  | .09 |  | -.06 |  | -.21* |  |
|  | Age x ROI | .06 |  | .09 |  | -.10 |  | -.18^†^ |  |
| Step 2 | DMThal |  | .63 |  | .64 |  | .63 |  | .64 |
|  | Age | -.23^†^ |  | -.21^†^ |  | -.22^†^ |  | -.16 |  |
|  | Sex | .14 |  | .15^†^ |  | .14 |  | .16^†^ |  |
|  | Single RT | .63*** |  | .64*** |  | .61*** |  | .65*** |  |
|  | ROI | .08 |  | .14 |  | -.01 |  | -.06 |  |
|  | Age x ROI | .06 |  | .08 |  | -.08 |  | -.16 |  |

β p-value: ^†^p<.10, *p<.05, **p<.01, ***p<.001; R^2^ p-value symbol represents statistical significance of R Square change.
