# Supplementary material for: Aging Brain from a Network Science Perspective: Something to Be Positive About?
Source: PLoS One. 2013 Nov 6;8(11):e78345. doi: 10.1371/journal.pone.0078345 (PMC3819386; doi:10.1371/journal.pone.0078345)
Supplement: Table S11 — Multiple linear regressions predicting SPWM accuracy from global and local efficiency in the fronto-parietal network. (DOCX) [file pone.0078345.s020.docx]

**Table S11**

| ROIs from the **Fronto-parietal Network**  DV: **SPWM accuracy (spatial working memory accuracy)** | | | | | | | | | |
| --- | --- | --- | --- | --- | --- | --- | --- | --- | --- |
|  |  | Global Efficiency | | | | Local Efficiency | | | |
|  |  | 250 | | 300 | | 250 | | 300 | |
|  |  | β | R^2^ | β | R^2^ | β | R^2^ | β | R^2^ |
| Step 1 |  |  | .12 |  | .12 |  | .12 |  | .12 |
|  | Age | .23^†^ |  | .23^†^ |  | .23^†^ |  | .23^†^ |  |
|  | Sex | .25^†^ |  | .25^†^ |  | .25^†^ |  | .25^†^ |  |
|  |  |  |  |  |  |  |  |  |  |
| Step 2 | SupPar |  | .13 |  | .13 |  | .14 |  | .12 |
|  | Age | .19 |  | .18 |  | .24 |  | .24 |  |
|  | Sex | .26^†^ |  | .26^†^ |  | .23^†^ |  | .25^†^ |  |
|  | ROI | .09 |  | .11 |  | -.16 |  | -.01 |  |
|  | Age x ROI | -.02 |  | -.01 |  | .18 |  | -.02 |  |
| Step 2 | LatOcc |  | .18 |  | .17 |  | .13 |  | .20^†^ |
|  | Age | .14 |  | .14 |  | .20 |  | .14 |  |
|  | Sex | .23^†^ |  | .25^†^ |  | .25^†^ |  | .22^†^ |  |
|  | ROI | .31^†^ |  | .27 |  | .12 |  | .38* |  |
|  | Age x ROI | -.11 |  | -.08 |  | -.06 |  | -.21 |  |
| Step 2 | LingFus |  | .17 |  | .18 |  | .12 |  | .16 |
|  | Age | .13 |  | .12 |  | .25 |  | .35* |  |
|  | Sex | .25^†^ |  | .24^†^ |  | .25^†^ |  | .25^†^ |  |
|  | ROI | .25 |  | .28^†^ |  | -.05 |  | -.24 |  |
|  | Age x ROI | -.04 |  | -.05 |  | .01 |  | .06 |  |

β p-value: ^†^p<.10, *p<.05, **p<.01, ***p<.001; R^2^ p-value symbol represents statistical significance of R Square change.
