# Supplementary material for: Aging Brain from a Network Science Perspective: Something to Be Positive About?
Source: PLoS One. 2013 Nov 6;8(11):e78345. doi: 10.1371/journal.pone.0078345 (PMC3819386; doi:10.1371/journal.pone.0078345)
Supplement: Table S12 — Multiple linear regressions predicting SPWM accuracy from global and local efficiency in the cerebellar and sub-cortical network. (DOCX) [file pone.0078345.s021.docx]

**Table S12**

| ROIs in **Cerebellum and Subcortical structures**  DV: **SPWM accuracy (spatial working memory accuracy)** | | | | | | | | | |
| --- | --- | --- | --- | --- | --- | --- | --- | --- | --- |
|  |  | Global Efficiency | | | | Local Efficiency | | | |
|  |  | 250 | | 300 | | 250 | | 300 | |
|  |  | β | R^2^ | β | R^2^ | β | R^2^ | β | R^2^ |
| Step 1 |  |  | .12 |  | .12 |  | .12 |  | .12 |
|  | Age | .23^†^ |  | .23^†^ |  | .23^†^ |  | .23^†^ |  |
|  | Sex | .25^†^ |  | .25^†^ |  | .25^†^ |  | .25^†^ |  |
|  |  |  |  |  |  |  |  |  |  |
| Step 2 | CBM |  | .14 |  | .14 |  | .13 |  | .15 |
|  | Age | .23 |  | .22 |  | .24^†^ |  | .29* |  |
|  | Sex | .29* |  | .28* |  | .27^†^ |  | .28* |  |
|  | ROI | .13 |  | .12 |  | .09 |  | .20 |  |
|  | Age x ROI | .04 |  | .04 |  | .02 |  | .01 |  |
| Step 2 | RedNuc |  | .13 |  | .13 |  | .13 |  | .12 |
|  | Age | .26 |  | .23 |  | .27^†^ |  | .24 |  |
|  | Sex | .24* |  | .24^†^ |  | .24^†^ |  | .25^†^ |  |
|  | ROI | .11 |  | .07 |  | .12 |  | .03 |  |
|  | Age x ROI | .04 |  | .05 |  | .04 |  | -.01 |  |
| Step 2 | DMThal |  | .18 |  | .16 |  | .13 |  | .15 |
|  | Age | .34* |  | .29^†^ |  | .20 |  | .21 |  |
|  | Sex | .28* |  | .27* |  | .25^†^ |  | .27* |  |
|  | ROI | .27^†^ |  | .19 |  | .09 |  | .16 |  |
|  | Age x ROI | .01 |  | .01 |  | .02 |  | -.01 |  |

β p-value: ^†^p<.10, *p<.05, **p<.01, ***p<.001; R^2^ p-value symbol represents statistical significance of R Square change.
